# The miR-644a/CTBP1/p53 axis suppresses drug resistance by simultaneous inhibition of cell survival and epithelial-mesenchymal transition in breast cancer

**DOI:** 10.18632/oncotarget.10489

**Published:** 2016-07-08

**Authors:** Umar Raza, Özge Saatci, Stefan Uhlmann, Suhail A Ansari, Erol Eyüpoğlu, Emre Yurdusev, Merve Mutlu, Pelin Gülizar Ersan, Mustafa Kadri Altundağ, Jitao David Zhang, Hayriye Tatlı Doğan, Gülnur Güler, Özgür Şahin

**Affiliations:** ^1^ Department of Molecular Biology and Genetics, Faculty of Science, Bilkent University, 06800 Ankara, Turkey; ^2^ Friedrich Miescher Institute for Biomedical Research, Maulbeerstrasse 66, 4058 Basel, Switzerland; ^3^ Department of Medical Oncology, Hacettepe University Cancer Institute, 06410 Ankara, Turkey; ^4^ Bäumlihofstrasse 429, 4125 Riehen, Switzerland; ^5^ Department of Pathology, Hacettepe University, 06410 Ankara, Turkey

**Keywords:** miRNAs, CTBP1, p53, EMT, therapy resistance

## Abstract

Tumor cells develop drug resistance which leads to recurrence and distant metastasis. MicroRNAs are key regulators of tumor pathogenesis; however, little is known whether they can sensitize cells and block metastasis simultaneously. Here, we report miR-644a as a novel inhibitor of both cell survival and EMT whereby acting as pleiotropic therapy-sensitizer in breast cancer. We showed that both miR-644a expression and its gene signature are associated with tumor progression and distant metastasis-free survival. Mechanistically, miR-644a directly targets the transcriptional co-repressor C-Terminal Binding Protein 1 (CTBP1) whose knock-outs by the CRISPR-Cas9 system inhibit tumor growth, metastasis, and drug resistance, mimicking the phenotypes induced by miR-644a. Furthermore, downregulation of CTBP1 by miR-644a upregulates wild type- or mutant-p53 which acts as a ‘molecular switch’ between G1-arrest and apoptosis by inducing cyclin-dependent kinase inhibitor 1 (p21, CDKN1A, CIP1) or pro-apoptotic phorbol-12-myristate-13-acetate-induced protein 1 (Noxa, PMAIP1), respectively. Interestingly, an increase in mutant-p53 by either overexpression of miR-644a or downregulation of CTBP1 was enough to shift this balance in favor of apoptosis through upregulation of Noxa. Notably, p53-mutant patients, but not p53-wild type ones, with high CTBP1 have a shorter survival suggesting that CTBP1 could be a potential prognostic factor for breast cancer patients with p53 mutations. Overall, re-activation of the miR-644a/CTBP1/p53 axis may represent a new strategy for overcoming both therapy resistance and metastasis.

## INTRODUCTION

Breast cancer is the most common malignancy and the second leading cause of cancer deaths among women [1]. Depending on breast cancer subtype [2], patients are treated with chemotherapy and/or targeted agents; however, intrinsic or acquired resistance is inevitable in almost all cases. As tumor cells develop *de novo* or acquired drug resistance, residing cancer cells undergo epithelial mesenchymal transition (EMT), evade from primary tumor site and metastasize to distant organs leading to death of the patients [3]. Therefore, it is necessary to identify novel targets which do not only inhibit tumor growth, but also sensitize refractory cells to therapy and prevent metastasis.

MicroRNAs (miRNA) are 20–22 nucleotide small non-coding RNAs which regulate gene expression post-transcriptionally by preferentially binding to the seed-matching sequence in the 3′-UTR of target mRNAs leading to either mRNA destabilization or degradation [4]. miRNAs have been classified as tumor suppressors or oncogenic ones depending on the phenotype they induce, the targets they modulate, and the tissue where they function [5, 6]. In this context, large number of oncogenic and tumor suppressor miRNAs have been shown to be associated with cancer progression, drug resistance or metastasis (reviewed in [7, 8]). However, little is known about miRNAs that can simultaneously regulate tumor proliferation and EMT whereby acting as therapy-sensitizer and metastasis blocker in breast cancer.

In this study, we identify miR-644a as a novel inhibitor of tumor cell proliferation and metastatic potential which acts as a pleotropic therapy sensitizer in breast cancer both *in vitro* and *in vivo*. These findings are further supported by the analysis of several breast cancer datasets. Mechanistically, we show that miR-644a directly targets transcriptional co-repressor CTBP1 and thereby upregulates p53 levels. We then show that this increased wild type or mutant p53 acts as a switch deciding on G1-arrest or apoptosis by inducing p21 or Noxa, respectively. Our *in silico* analyses propose CTBP1 as an important predictor for survival of breast cancer patients with p53 mutation. These results suggest that the re-activation of miR-644a/CTBP1/p53 axis may represent a new target to overcome breast cancer progression, therapy resistance, and metastasis.

## RESULTS

### miR-644a inhibits proliferation, promotes apoptosis, and its expression or gene signature correlates with tumor progression in breast cancer

**Figure 1:**
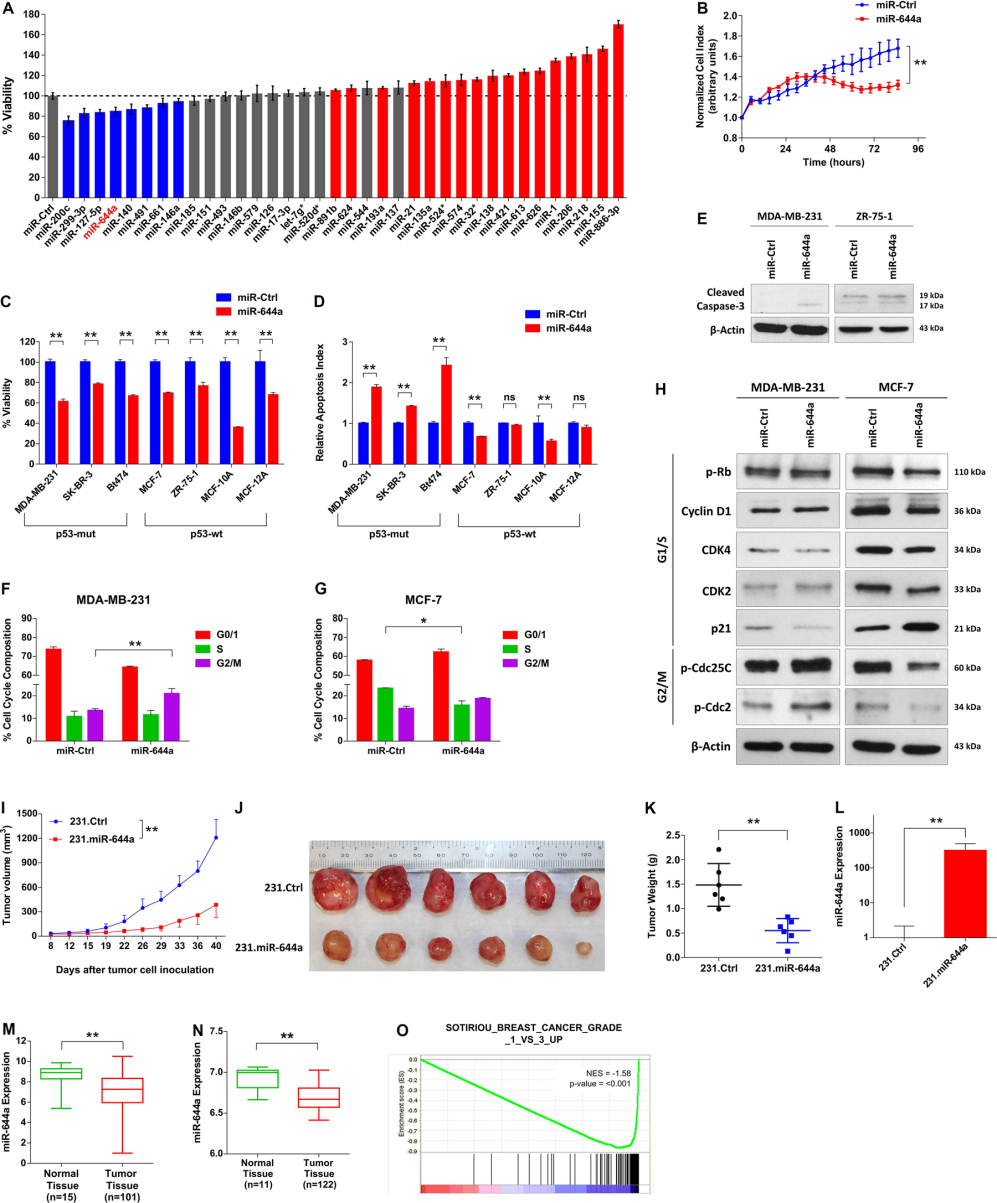
miR-644a reduces the viability of breast cancer cells *in vitro* and *in vivo* and miR-644a expression or its gene signature is associated with tumor progression in breast cancer (**A**) miRNA mimic cell viability screen on MDA-MB-231 human breast cancer cell line comprising of 35 different miRNAs, with miR-200c as a positive control. The cells were transfected with 20 nM of mimics for 48 hours, and viability was measured using Cell titer Glo. Color coding of the bars depicts the effect of each miRNA on cell viability (blue: decreasing viability, red: increasing viability, gray: no effect on viability). (**B**) Real time growth of MDA-MB-231 cells transiently transfected with either a control miRNA (miR-Ctrl) or miR-644a, monitored using an RTCA (real-time cell analyzer) assay. (**C**) Effect of miR-644a overexpression on proliferation of 5 breast cancer cell lines and 2 normal breast cell lines transfected with either miR-Ctrl or miR-644a. *n* = 4. (**D**) Changes in the apoptotic index based on Caspase-3 cleavage in cells from (C). *n* = 4. (**E**) Western Blot Analysis showing the levels of cleaved Caspase-3 in p53-*mut* MDA-MB-231 (left) and p53-*wt* ZR-75-1 cells (right) after 72 hours transfection with either miR-Ctrl or miR-644a. (**F** and **G**) Flow cytometric analysis of cell cycle in cells transfected with miR-Ctrl or miR-644a showing G2/M arrest in miR-644a transfected MDA-MB-231 cells (F) and G1 arrest in miR-644a transfected MCF-7 cells (G). (**H**) Western Blot Analysis showing the levels of cell cycle proteins related to G1/S (pRb, Cyclin D1, CDK4, CDK2 and p21) and G2/M transition (p-Cdc25C and p-Cdc2) in p53-*mut* MDA-MB-231 (left) and p53-*wt* MCF7 cells (right) after 48 hours transfection with either miR-Ctrl or miR-644a. (**I**) Tumor progression in xenografts generated by orthotropic subcutaneous injection of MDA-MB-231 cells stably expressing either a non-silencing control (231.Ctrl) or miR-644a (231.miR-644a) into nude mice. *n* = 6. (**J**) Representative images of tumors collected from xenografts of (I) on day 40. (**K**) Tumor weights in xenografts from (I) at day 40. (**L**) qRT-PCR analysis showing average miR-644a expression in 231.Ctrl and 231.miR-644a tumors collected from xenografts of (I) on day 40. *n* = 3. (**M**) miR-644a expression in 101 breast tumor tissues and 15 normal tissues from GSE45666 depicted as box-plot showing the median expression in all patients. (**N**) miR-644a expression in 122 breast tumor tissue and 11 normal tissue samples from GSE58606. (**O**) Enrichment plots of patients from GSE58644 (*n* = 320) with high or low miR-644a signature score. Genes up-regulated as breast tumors progress through histologic grade 3 were enriched in patients with low miR-644a signature score. Statistical significance was indicated (*p < 0.05; **p < 0.01; ns, not significant). Column data represent mean ± SD. Box-plots depict median number and the 25^th^ to 75^th^ quartiles. Upper and lower whiskers represent the minimum and maximum values in the corresponding group. This applies to all figures shown.

To identify novel miRNAs regulating proliferation in breast cancer, we performed a small scale miRNA mimic cell viability screen entailing 35 miRNAs in MDA-MB-231 human breast cancer cell line (Figure 1A). As a positive control we used miR-200c, which was previously reported as a tumor suppressor miRNA by us [9] and others [10, 11]. Out of three most promising potential tumor suppressor miRNAs besides miR-200c, miR-299–3p and miR-127–5p have been reported as tumor suppressors in different cancer types [12, 13]. The other one, miR-644a, has not been characterized in the context of breast cancer. Real time cell analyzer (RTCA) assay further confirmed inhibitory role of miR-644a in viability of MDA-MB-231 cells (Figure 1B). Furthermore, miR-644a reduced viability of other cell lines representing different breast cancer subtypes and two normal breast cell lines, MCF-10A and MCF-12A, (Figure 1C).

Upon miR-644a overexpression, breast cancer cell lines with p53 mutation (p53-*mut*) only underwent apoptosis evidenced by increased cleaved caspase-3 (Figure 1D and 1E) by inducing G2/M arrest characterized by increased phosphorylation of G2/M-arrest markers Cdc2 and Cdc25C (Figure 1F and 1H). In contrast, miR-644a overexpression in p53-*wt* MCF-7 cells resulted in G1 arrest with decreased expression of G1/S transition proteins and increased expression of CDK inhibitor p21, which leads to reduced phosphorylation of Rb protein (Figure 1G and 1H).

To validate our findings *in vivo*, we engineered MDA-MB-231 cell line (referred to herein as 231.Ctrl) with lentiviral-transduced miR-644a (referred to herein as 231.miR-644a) (Supplementary Figure S1A), and observed a delayed and significantly decreased tumor growth (Figure 1I). Correspondingly, tumors collected from the 231.miR-644a group showed high levels of miR-644a expression (Figure 1L), were substantially smaller and weighed less (Figure 1J and 1K) further confirming the tumor suppressive role of miR-644a in breast cancer.

To elucidate the pathological relevance of miR-644a, we examined the expression of miR-644a in publicly available expression datasets GSE45666 and GSE58606 [14, 15], and observed significantly lower miR-644a levels in tumors as compared to normal tissues (Figure 1M and 1N). Besides breast cancer, melanoma and osteosarcoma cell lines also had lower miR-644a levels compared to their normal counterparts (Supplementary Figure S1B and S1C). In addition to gene-level analysis, we also performed gene expression profiling to derive a miR-644a signature, and used this signature to elucidate miR-644a-induced changes in a more global manner. To this end, we forced expression of miR-644a using mimics in three breast cancer cell lines representing different subtypes of breast cancer (Supplementary Figure S2A). We then collected commonly up- and down-regulated genes among these three cell lines to create a ‘miR-644a signature’ (Supplementary Table S1). Gene Ontology (GO) term analysis revealed association of miR-644a signature with biological processes contributing to tumor progression, such as cell cycle, apoptosis, actin cytoskeletal organization and cell adhesion (Supplementary Figure S2B). Furthermore, Gene Set Enrichment Analysis (GSEA) of GSE58644 dataset [16] showed a significant correlation between miR-644a gene signature and tumor progression in breast cancer patients. Notably a gene set containing genes downregulated in lobular carcinoma compared to normal lobular breast cells were enriched in patients with high miR-644a signature scores (Supplementary Figure S1D). Moreover, genes associated with histologic grade 1 and grade 3 in breast cancer patients were significantly enriched in high and low miR-644a signature scorers, respectively (Figure 1O and Supplementary Figure S1E). Consistent with this observation, in the same dataset, miR-644a signature score was lower in tumors with a more aggressive disease state characterized by higher tumor grade and stage (Supplementary Figure S1F and S1G). Finally, miR-644a status was correlated with the formation and progression of not only breast cancer, but also of a variety of cancers, including melanoma, liver, lung and ovarian cancers (Supplementary Table S2), all supporting the tumor suppressive roles of miR-644a.

### miR-644a inhibits metastasis and correlates with distant metastasis-free survival in patients

**Figure 2:**
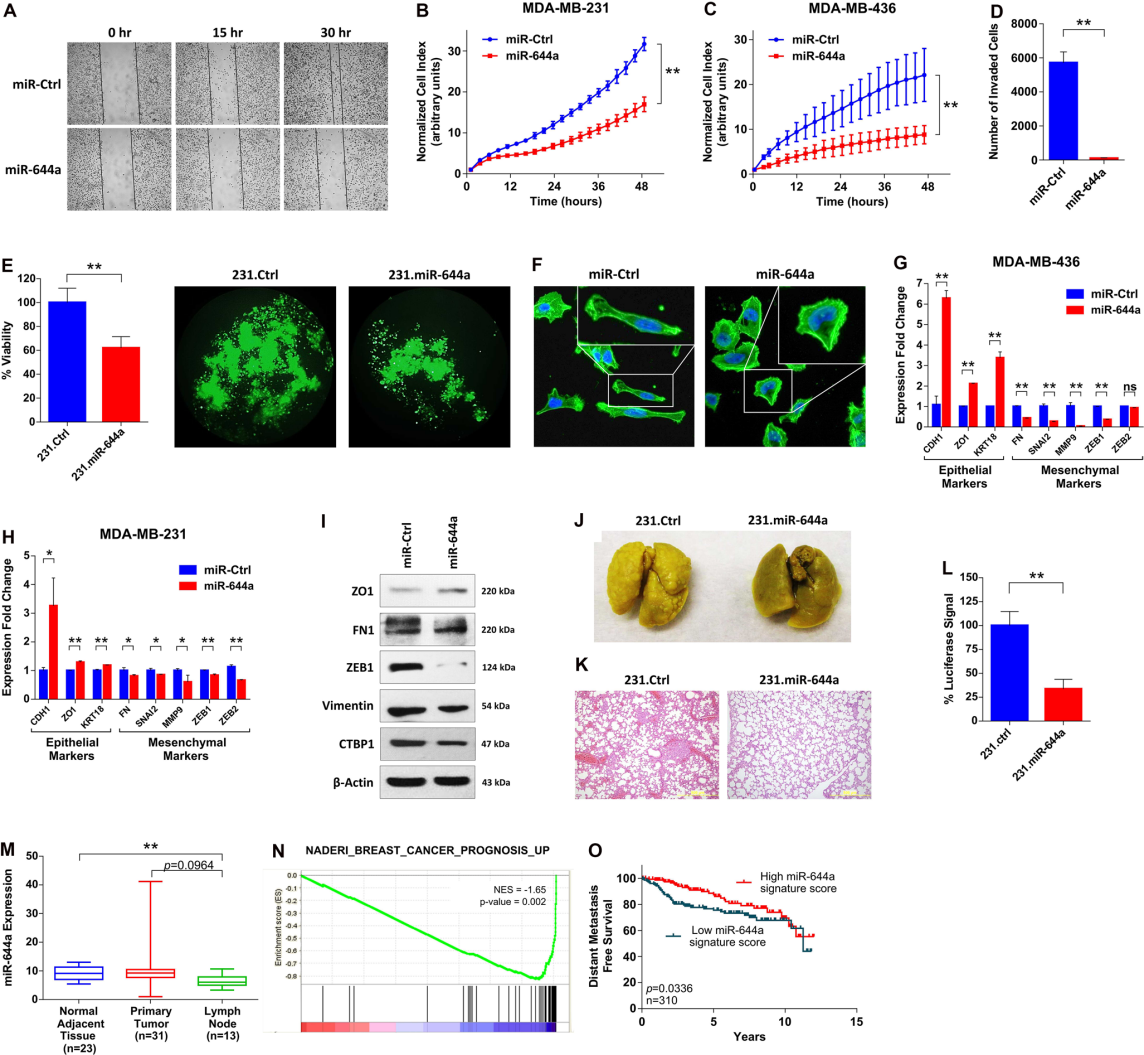
miR-644a inhibits metastasis, and its expression or gene signature is associated with metastasis of breast cancer patients (**A**) Wound healing assay of MDA-MB-231 cells transfected with miR-Ctrl or miR-644a. Cells were scratched after 48 hours of transfection, and images were taken with 4× Magnification at 0, 15 and 30 hours after transfection. (**B** and **C**) Real-time migration of MDA-MB-231 (B) and MDA-MB-436 (C) cells transfected with either miR-Ctrl or miR-644a, monitored using an RTCA assay. (**D**) Number of invaded cells transfected with miR-Ctrl or miR-644a using Matrigel invasion assay. *n* = 3. (**E**) Viability of 231.Ctrl and 231.miR-644a cells grown in anchorage-independent conditions for 7 days, quantified by WST-1 assay (left) together with their fluorescence microscopy images with 10X magnification (right). (**F**) Fluorescence microscopy images of MD-MB-231 cells transfected with miR-Ctrl or miR-644a. Cell nuclei and filamentous actin were stained with 4,6-diamidino-2-phenylindole (DAPI) and Alexa Fluor 488 phalloidin, respectively. Images were taken after 72 hours of transfection with 20X magnification. Boxes at upper right corners of the images show cell morphology with higher resolution. (G and H) qRT-PCR analysis of epithelial and mesenchymal marker gene expression in MDA-MB-436 (**G**) and MDA-MB-231. (**H**) Cells transfected with miR-Ctrl or miR-644a. *n* = 3. (**I**) Western blot analysis of epithelial and mesenchymal marker expression in MDA-MB-231 cells transfected with miR-Ctrl or miR-644a. (**J**) Representative images of lungs collected from nude mice injected intravenously with 231.Ctrl or 231.miR-644a cells. Mice were sacrificed at week 7 and lungs were fixed in Bouin's Solution. (**K**) Hematoxylin and eosin staining of metastatic nodules in lungs from (J). (**L**) Luciferase signal coming from metastatic nodules in lungs of (J) as quantified by a luciferase assay. (**M**) miR-644a expression in 23 normal tissue, 31 primary tumor (IDC) and 13 lymph node metastasis tissues in GSE38167 depicted as box-plot. (**N**) Enrichment plot of patients from GSE58644 (*n* = 320) with high or low miR-644a signature score. Genes expressed higher in breast cancer patients with poor outcome as compared to those with good outcome were enriched in patients expressing low levels of miR-644a signature score. (**O**) Kaplan Meier survival curve representing the percentage distant metastasis-free survival in breast cancer patients based on miR-644a signature score median expression levels in GSE58644 (*n* = 310).

Since we observed enrichment of biological processes relevant to metastasis (Supplementary Table S3), we wanted to examine the effects of miR-644a on the metastatic potential of breast tumors. Transient or stable miR-644a overexpression significantly inhibited migration and invasion of MDA-MB-231 cells as assessed by wound healing, real-time migration and trans-well matrigel invasion assays (Figure 2A–2D) and reduced anchorage independent growth (Figure 2E). Furthermore, cells overexpressing miR-644a showed rearrangements of actin cytoskeletal structures from a mesenchymal to an epithelial-like state (Figure 2F) which is further confirmed by upregulated epithelial markers and downregulated mesenchymal markers both at mRNA and protein level upon miR-644a overexpression (Figure 2G–2I). Further, we tested the metastatic potential of 231.miR-644a cells in nude mice with tail-vein metastasis assay. Bouin's fixation of lungs and Hematoxylin and Eosin (H&E) staining indicated less colonization of 231.miR-644a cells to lungs as compared to 231.Ctrl cells (Figure 2J and 2K). As these cells are stably labelled with luciferase, we measured luciferase activity of lung lysates, and showed that substantially less number of cells reached metastasized to the lungs when miR-644a is expressed (Figure 2L). These data suggest that miR-644a also inhibits metastasis *in vivo*.

Then, to validate these *in vitro* and *in vivo* findings in human patient datasets, we first examined GSE38167 dataset [17], and found that expression of miR-644a is lower in primary tumors compared to normal adjacent tissues, and even further decreased in lymph node metastases (Figure 2M). This observation was further validated by GSEA which revealed an enrichment of genes whose expression in primary tumors of estrogen receptor positive (ER+) breast cancer positively correlates with developing distant metastases are enriched in patients with low miR-644a signature scores from GSE58644 (Supplementary Table S3). Moreover, miR-644a expression was found to be negatively associated with metastasis in cancers other than breast as well (Supplementary Figure S1H and S1I; Supplementary Table S3). Finally, we showed an enrichment of genes associated with poor outcome in patients having low miR-644a signature scores (Figure 2N). Consistent with this, breast cancer patients with high miR-644a signature scores have significantly longer distant-metastasis-free survival (Figure 2O). Overall, our data suggest that miR-644a is a novel tumor suppressor that is likely to be involved in progression and metastasis of multiple cancer types including breast cancer.

### miR-644a is a pleiotropic therapy sensitizer in breast cancer

**Figure 3:**
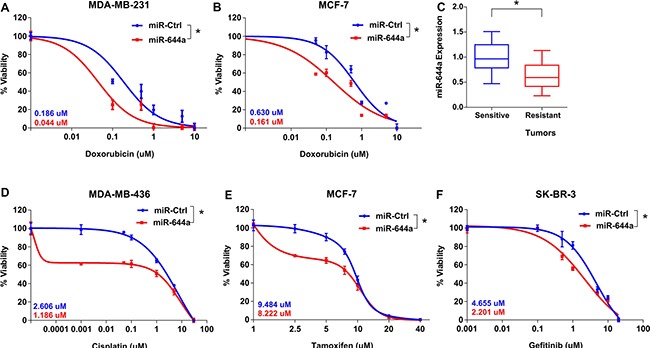
miR-644a overexpression acts as a therapy sensitizer in breast cancer cells and its expression correlates with doxorubicin resistance *in vivo* xenografts (**A** and **B**) Effect of miR-644a overexpression on the response of MDA-MB-231 (A) and MCF-7 (B) cells to doxorubicin. (**C**) qRT-PCR analysis of miR-644a expression in xenografts sensitive or resistant to doxorubicin. Nude mice were subcutaneously injected with MDA-MB-231 cells and treated with doxorubicin. Among the treated mice, sensitive and resistant tumors were selected based on changes in tumor volumes upon successive drug treatments. *n* = 4. (**D**–**F**) Effect of miR-644a overexpression on the response of MDA-MB-436 cells to cisplatin (D), response of MCF-7 cells to tamoxifen (E), and response of SKBR-3 cells to gefitinib (F). IC50 values for each condition are given on the left bottom corners of each curve with a color code.

Since miR-644a inhibits both breast cancer cell survival and EMT, we hypothesized that it might also work as a therapy sensitizer. To test this hypothesis, we did GSEA with gene sets related to drug sensitivity and resistance. We observed that genes associated with doxorubicin (topoisomerase II inhibitor) and cisplatin (DNA cross linking agent; promising therapy for BRCA1/2 mutated/deficient tumors) resistance in gastric cancer cell lines and patients, respectively were significantly enriched in patients with low miR-644a signature scores (Supplementary Table S3). Indeed, miR-644a significantly sensitized p53-*mut* MDA-MB-231 and p53-*wt* MCF-7 cells to doxorubicin and *BRCA1*-mutated MDA-MB-436 breast cancer cells to cisplatin *in vitro* (Figure 3A, 3B and 3D). Furthermore, significantly higher miR-644a levels were observed in doxorubicin sensitive tumors developed *in vivo* as compared to resistant ones (Figure 3C), further supporting that miR-644a may play a role in chemotherapy resistance also *in vivo*.

In addition to chemotherapy agents, we found that patients with higher miR-644a signature scores have enhanced sensitivity to tamoxifen (Supplementary Table S3), which is the mainstay targeted therapy for ER+ breast cancer patients for over 40 years [18]. We demonstrated that forced miR-644a expression sensitizes cells to tamoxifen in ER+ MCF-7 cells *in vitro* (Figure 3E). Similarly, we saw that genes downregulated in gefitinib resistant non-small cell lung cancer cells undergoing prominent growth arrest and apoptosis upon treatment with an irreversible EGFR inhibitor, CL-387,785 [19], were enriched in low miR-644a signature scorers (Supplementary Table S3). We validated this in EGFR overexpressing SK-BR-3 breast cancer cells, where miR-644a overexpression significantly sensitized cells to gefitinib (Figure 3F). Overall, multiple lines of evidence support the notion that miR-644a may be a pleiotropic sensitizer for both chemo- and targeted-therapy.

### CTBP1 is a direct target of miR-644a

**Figure 4:**
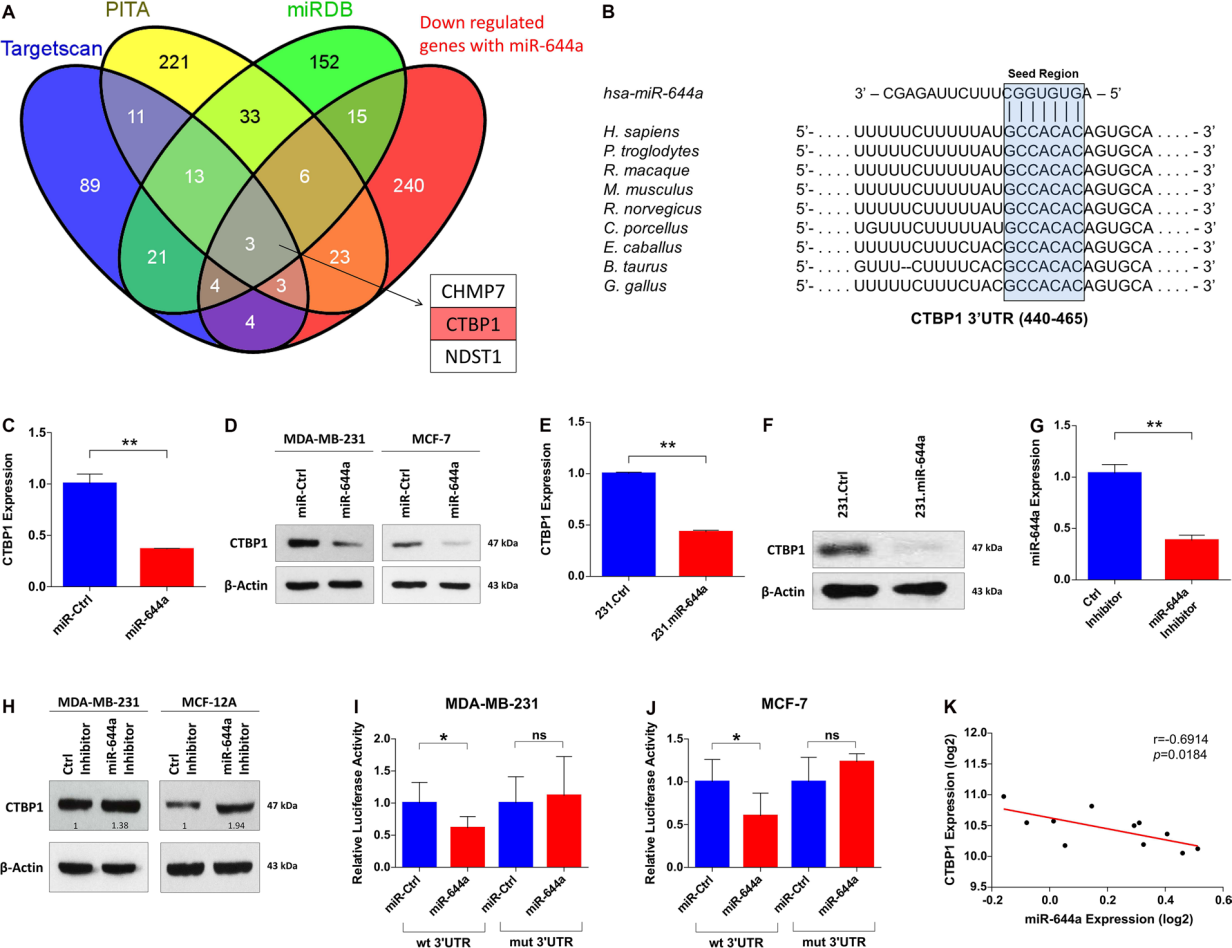
CTBP1 is a direct target of miR-644a (**A**) Venn diagram for the combinatorial target prediction analysis. List of genes downregulated by miR-644a in the microarray analysis was combined with genes predicted to be miR-644a targets by three different target prediction algorithms namely TargetScan (blue), PITA (yellow) and miRDB (green). Three genes that are common in all four groups were depicted, with CTBP1 highlighted in red. (**B**) Schematic diagram showing miR-644a binding site in *CTBP1* 3′-UTR (453–460) in different species including human. (**C** and **D**) Confirmation of CTBP1 downregulation by miR-644a overexpression at transcript and protein levels with qRT-PCR analysis in MDA-MB-231 (C) and with Western Blot analysis in MDA-MB-231 (left) and MCF-7 (right) (D) cells transfected with either miR-Ctrl or miR-644a. *n* = 3 for (C). (**E** and **F**) qRT-PCR (E) and Western Blot (F) analysis of CTBP1 expression in 231.Ctrl and 231.miR-644a cells confirming downregulation of CTBP1 in transcript and protein levels in cells stably expressing miR-644a. *n* = 3 for (E). (**G**) qRT-PCR analysis of miR-644a expression in MDA-MB-231 cells transfected with either a control Inhibitor (Ctrl Inhibitor) or miR-644a Inhibitor. *n* = 3. (**H**) Western Blot Analysis showing the levels of CTBP1 in MDA-MB-231 and MCF-12A cells transfected with either Ctrl Inhibitor or miR-644a Inhibitor. (**I** and **J**) Luciferase activity of a reporter construct fused with either a *wt* or *mut CTBP1* 3′-UTR in MDA-MB-231 (I) and MCF-7 (J) cells transfected with miR-Ctrl or miR-644a. *n* = 5. (**K**) Expression of miR-644a negatively correlates with CTBP1 expression in 9 breast cancer cell lines and 2 normal breast cell lines from GSE40059.

To identify the targets of miR-644a mediating these observed effects, we combined the list of genes downregulated upon miR-644a overexpression in our microarray analysis with targets of miR-644a predicted by three target prediction algorithms. This stringent analysis resulted in 3 genes: *CTBP1*, *CHMP7*, and *NDST1* (Figure 4A). We found CTBP1 as the most promising candidate since it is an established transcriptional co-repressor which preferentially represses the transcription of tumor suppressor genes and promotes tumor growth *via* playing pivotal roles in tumor pathogenesis [20–22].

Sequence analysis revealed that human *CTBP1* 3′-UTR has one binding site of miR-644a between nucleotides 453–460 (Figure 4B). Transient as well as stable overexpression of miR-644a significantly downregulated CTBP1 mRNA and protein levels in both MDA-MB-231 and MCF-7 cells (Figure 4C–4F). Inversely, miR-644a inhibition in MDA-MB-231 and MCF-12A cells by using hairpin inhibitors upregulated CTBP1 levels (Figure 4G and 4H). We confirmed CTBP1 as a direct target of miR-644a by measuring luciferase expression from *CTBP1* 3′-UTR constructs with or without mutation (Supplementary Figure S3A) in MDA-MB-231 and MCF-7 cells upon miR-644a transfection. In both cell lines, miR-644a overexpression significantly repressed luciferase expression when co-transfected with Wild type 3′-UTR expressing vector, but not in the case of mutated 3′-UTR expressing vector (Figure 4I and 4J). Additionally, through analyzing mRNA and miRNA expression profile dataset GSE40059 [23], we observed an inverse correlation between miR-644a and CTBP1 expression in 11 different breast cancer cell lines (Figure 4K). Overall, these data confirm that CTBP1 is a direct target of miR-644a.

### Loss of CTBP1 mimics tumor- and metastasis-suppressive roles of miR-644a *in vitro* and *in vivo*

**Figure 5:**
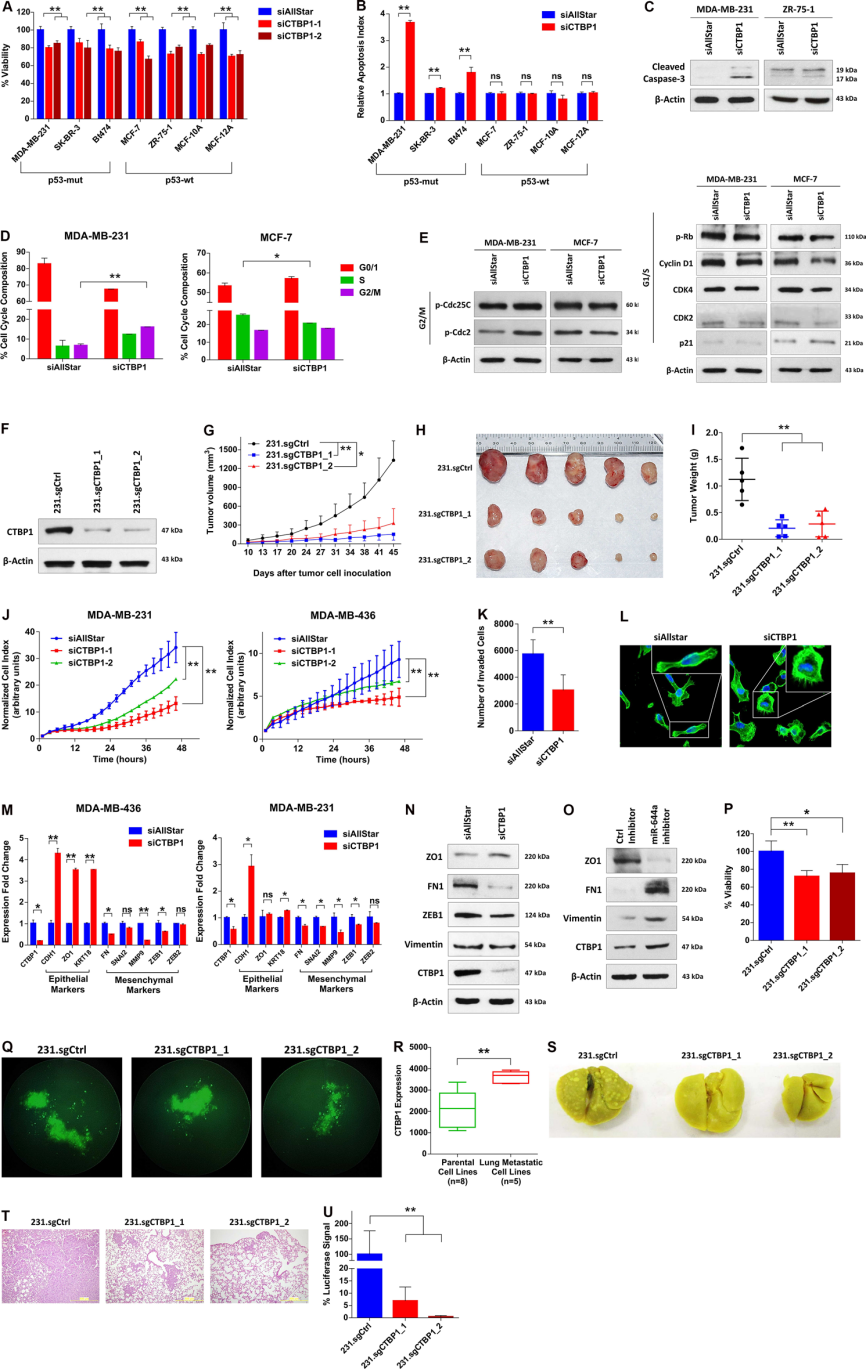
Loss of CTBP1 inhibits cell viability, tumor growth, migration and invasion *in vitro*, and inhibits tumor progression and metastasis *in vivo* (**A**) Effect of CTBP1 knockdown on proliferation of cell lines previously used to test the effects of miR-644a overexpression on proliferation as in Figure 1C. Cells were transfected with either a non-targeting siRNA control (siAllStar) or different CTBP1 targeting siRNAs (siCTBP1–1, siCTBP1–2). *n* = 4. (**B**) Changes in the apoptotic index based on Caspase-3 cleavage in cells from (A) transfected with siAllStar or siCTBP1-Pool. *n* = 4. (**C**) Western Blot Analysis showing the levels of cleaved Caspase-3 in p53-*mut* MDA-MB-231 (left) and p53-*wt* MCF7 cells (right) after 72 hours transfection with siAllStar or siCTBP1-Pool. (**D**) Flow cytometric analysis of cell cycle in cells transfected with siAllStar or siCTBP1-Pool showing G2/M arrest in siCTBP1-Pool transfected MDA-MB-231 cells (left) and G1 arrest in siCTBP1-Pool transfected MCF-7 cells (right). (**E**) Western Blot analysis showing the levels of cell cycle proteins related to G2/M arrest (p-Cdc25C and p-Cdc2) (left) and G1/S (pRb, Cyclin D1, CDK4, CDK2 and p21) (right) transition in p53-*mut* MDA-MB-231 and p53-*wt* MCF7 cells after 48 hours transfection with siAllStar or siCTBP1-Pool. (**F**) Western Blot Analysis of CTBP1 levels in MDA-MB-231.luc cells stably expressing either a non-targeting sgRNA (231.sgCtrl) or different CTBP1 targeting sgRNAs (231.sgCTBP1_1, 231.sgCTBP1_2) confirming stable knock-out of CTBP1. (**G**) Tumor progression in xenografts generated by orthotopic subcutaneous injection of 231.sgCtrl, 231.sgCTBP1_1 or 231.sgCTBP1_2. *n* = 5. (**H**) Representative images of tumors collected from xenografts of (G) on day 45. (**I**) Tumor weights in xenografts from (G) at day 45. (**J**) Real time migration of MDA-MB-231 (left) and MDA-MB-436 (right) cells transfected with siAllStar, siCTBP1–1 or siCTBP1–2, monitored using an RTCA assay. (**K**) Number of invaded cells transfected with siAllStar or siCTBP1-Pool using Matrigel invasion assay. *n* = 3. (**L**) Fluorescence microscopy images of MDA-MB-231 cells transfected with either siAllStar or siCTBP1-Pool. Cell nuclei and filamentous actin were stained with DAPI and Alexa Fluor 488 phalloidin, respectively. Images were taken after 72 hours of transfection with 20× magnification. Boxes at the upper right corners of the images show cell morphology with higher resolution. (**M**) qRT-PCR analysis of epithelial and mesenchymal marker gene expression in MDA-MB-436 (left) and MDA-MB-231 (right) cells transfected with either siAllStar or siCTBP1-Pool. *n* = 3. (**N**) Western blot analysis of epithelial and mesenchymal marker expression in MDA-MB-231 cells transfected with either siAllStar or siCTBP1-Pool. (**O**) Western blot analysis of epithelial and mesenchymal marker expression in MCF-12A cells transfected with either Ctrl inhibitor or miR-644a inhibitor. (**P** and **Q**) Viability of 231.sgCtrl, 231.sgCTBP1_1 and 231.sgCTBP1_2 cells grown in anchorage-independent conditions for 7 days, quantified by WST-1 assay (P) together with their fluorescence microscopy images with 10× magnification (Q). (**R**) CTBP1 expression in parental MDA-MB-231 cells, its single-cell-derived progenies (SCPs) and *in vivo* isolated sub-lines with different lung metastatic capabilities from GSE2603 (*n* = 19). (**S**) Representative images of lungs collected from nude mice injected intravenously with 231.sgCtrl, 231.sgCTBP1_1 or 231.sgCTBP1_2. Mice were sacrificed at week 7 and lungs were fixed in Bouin's Solution. (**T**) Hematoxylin and eosin staining of metastatic nodules in lungs from (S). (**U**) Luciferase signal coming from metastatic nodules in lungs of (S) as quantified by a luciferase assay.

To validate that CTBP1 is a major functional target of miR-644a, we first knocked down CTBP1 with two different siRNA sequences (Supplementary Figure S4A and S4B), and examined the effect on viability, apoptosis and cell cycle. We observed a significant reduction in the viability of all tested cell lines upon CTBP1 knockdown (Figure 5A). Interestingly, CTBP1 downregulation also mimicked miR-644a overexpression in increasing apoptotic cell death and inducing cleaved caspase-3 exclusively in p53-*mut* breast cancer cell lines, but not in p53-*wt* cell lines (Figure 5B and 5C). Moreover, cell cycle and western blot analysis confirmed a G2/M arrest in p53-*mut* MDA-MB-231 cells while a G1 arrest was observed in p53-*wt* MCF-7 cells upon CTBP1 knockdown similar to the effect of miR-644a (Figure 5D and 5E). For *in vivo* validation, we generated two independent CRISPR-Cas9 mediated CTBP1 knock-outs in MDA-MB-231 cells (referred to herein as 231.sgCTBP1_1 and 231.sgCTBP1_2) (Supplementary Figure S3B and S3C). Both cell lines showed efficient downregulation of CTBP1 (Figure 5F), and exhibited delayed and significantly decreased tumor growth and tumor size in nude mice (Figure 5G–5I). We could also replicate these findings using an shRNA construct against CTBP1 (Supplementary Figure S5A–S5D).

Next, we tested if the loss of CTBP1 is able to mimic the effects of miR-644a on tumor metastasis. CTBP1 knockdown significantly inhibited migration and invasion of both MDA-MB-231 and MDA-MB-436 cells (Figure 5J and 5K), and induced a more epithelial-like state (Figure 5L–5N). Inversely, CTBP1 upregulation upon miR-644a inhibitor transfection promoted mesenchymal-like state (Figure 5O). Furthermore, CTBP1 knock-outs by sgRNAs or knockdown by shRNA resulted in a significant reduction in anchorage-independent growth (Figure 5P and 5Q; Supplementary Figure S5E). Re-analysis of the GSE2603 dataset [24] revealed that *in vivo* sub-lines of MDA-MB-231 with enhanced lung metastatic ability express substantially higher CTBP1 levels compared to parental cells with limited lung metastatic potential (Figure 5R). We then set to test the effect of CTBP1 in lung metastasis, and demonstrated markedly less colonization of 231.sgCTBP1_1 and 231.sgCTBP1_2 cells to lungs compared to 231.sgCtrl cells as demonstrated by Bouin's fixation and H&E staining of lungs and luciferase assay with lung lysates (Figure 5S–5U).

To elucidate the role of CTBP1 in tumor progression and metastasis in breast cancer patients, we analyzed several patient datasets. In GSE4922 [25] and GSE58644 datasets, CTBP1 levels were found to be correlated with higher tumor size and incidence of developing distant metastasis (Supplementary Figure S5F and S5G). Finally, we retrieved data form the Molecular Taxonomy of Breast Cancer International Consortium (METABRIC) project [26], and performed targeted analysis. We found that Stage 2 and 3 breast cancers where cancer starts to spread to nearby lymph nodes feature higher CTBP1 levels compared to Stage 1 breast cancer which has no or only microscopic invasion smaller than 1 mm [27]. There was also a significant increase in CTBP1 levels in Stage 4 tumors, characterized by the presence of metastases to organs other than breast (Supplementary Figure S5H). In accordance with the tumor stage, patients with 20 or more positive lymph nodes had higher CTBP1 in their primary tumors suggesting a role of CTBP1 in promoting lymph node metastasis (Supplementary Figure S5I). All of these findings suggest that CTBP1 inhibition mimics miR-644a overexpression in inhibition of breast tumor growth and metastasis.

### CTBP1 is a major functional target of miR-644a mediating drug resistance and EMT

**Figure 6:**
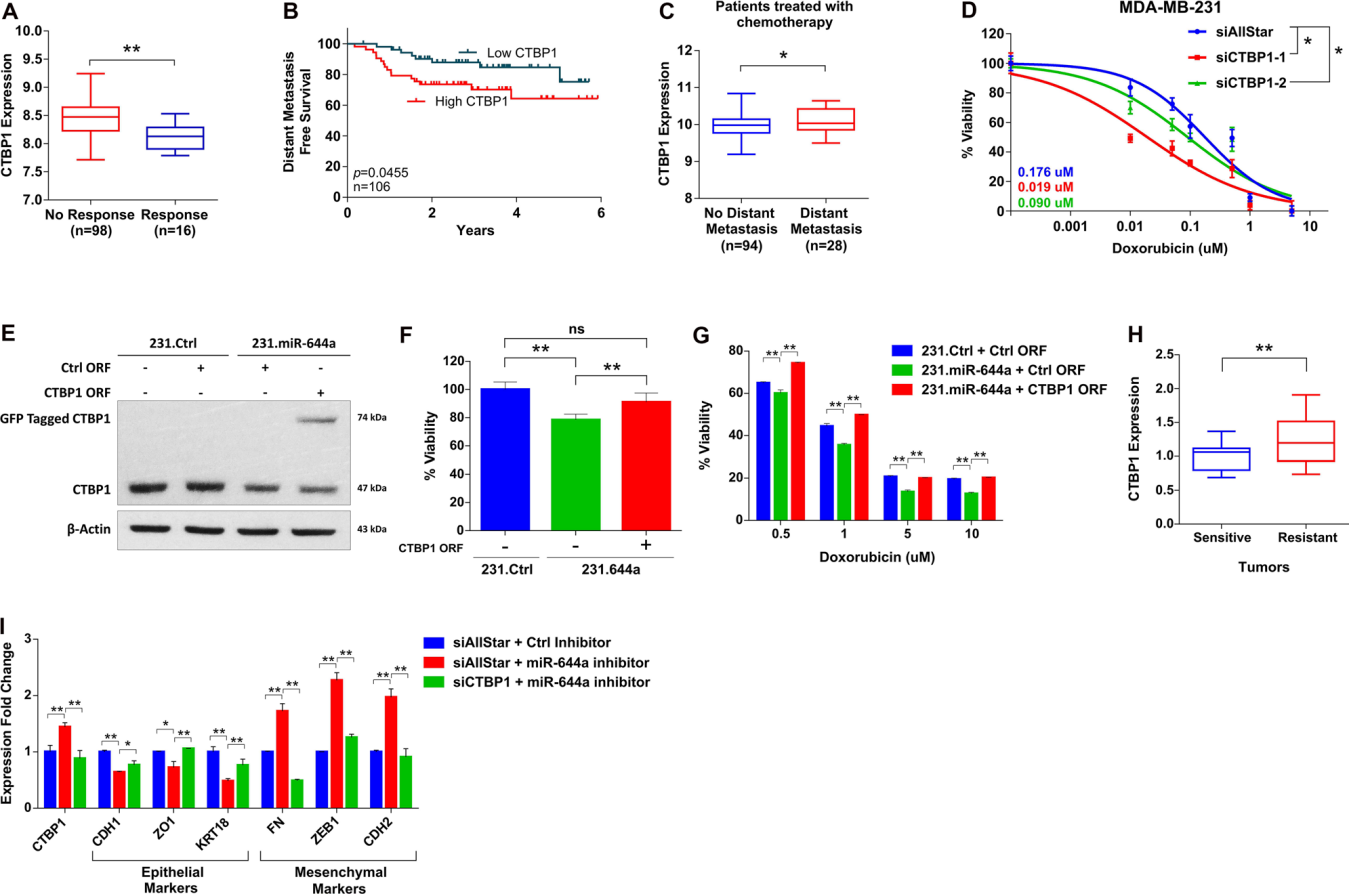
CTBP1 expression correlates with response to chemotherapy, and its loss sensitizes to chemotherapy and enhances epithelial-like state (**A**) CTBP1 expression in anthracycline treated breast cancer patients from GSE16446 with no response to treatment (*n* = 98) or pathologic complete response (*n* = 16). (**B**) Kaplan Meier survival curve representing the percentage distant metastasis-free survival in breast cancer patients treated with anthracyclines based on CTBP1 median expression levels in GSE16446 dataset (*n* = 106). (**C**) CTBP1 expression in patients with no distant metastasis (*n* = 94) or with distant metastasis (*n* = 28) among breast cancer patients treated with chemotherapy from GSE58644. (**D**) Effect of CTBP1 knockdown on the response of MDA-MB-231 cells to doxorubicin. Cells were transfected with siAllStar, siCTBP1–1 or siCTBP1–2 and treated with increasing concentrations of doxorubicin. *n* = 4. (**E** and **F**) 231.Ctrl or 231.miR644a cells were transfected with Ctrl open reading frame (ORF) or CTBP1 ORF in mentioned combination to rescue the CTBP1 expression. Western Blot analysis showing rescue of CTBP1 expression in MDA-MB-231 (E). Effect of CTBP1 rescue on viability of MDA-MB-231 cells (F). (**G**) Bar graph showing the effect of CTBP1 rescue as shown in (F) on the response of MDA-MB-231 to 4 different doses of doxorubicin. *n* = 4. (**H**) qRT-PCR analysis of CTBP1 expression in xenografts sensitive or resistant to doxorubicin that were previously used to test the changes in miR-644a levels upon drug resistance (Figure 3C). *n* = 3. (**I**) qRT-PCR analysis of epithelial and mesenchymal marker gene expression in MCF-12A cells upon CTBP1 rescue by miR-644a inhibitor and further knockdown by siCTBP1.

We tested if CTBP1 mediates the effect of miR-644a on drug resistance. *In silico* analysis of GSE16446 dataset [28–30] showed significantly lower levels of CTBP1 in patients with pathologic complete response (pCR) compared to patients without complete response against anthracycline treatment (Figure 6A). In the same dataset, low CTBP1 level was associated with better outcome in terms of distant metastasis-free survival (DMFS) (Figure 6B). In GSE58644, among patients treated with chemotherapy, distant metastasis incidence rate was significantly higher in patients with high CTBP1 (Figure 6C). All these suggest an important role of CTBP1 in tumor recurrence in chemotherapy treated patients.

We then tested if we can mimic the effect of miR-644a on chemotherapy response by knocking down CTBP1. We observed that knockdown of CTBP1 sensitized MDA-MB-231 cells to doxorubicin (Figure 6D). Furthermore, rescuing CTBP1 levels along with miR-644a overexpression (Figure 6E) not only increased the viability of MDA-MB-231 cells (Figure 6F), but also rendered these cells less sensitive to doxorubicin treatment compared to those cells in which only miR-644a was overexpressed (Figure 6G). Furthermore, we observed a significantly lower levels of CTBP1 in doxorubicin sensitive tumors compared to resistant tumors of the mouse models we developed (shown in Figure 3C), which is exactly the opposite of miR-644a levels in these tumors (Figure 6H). Finally, CTBP1 rescue *via* miR-644a inhibitor transfection promoted mesenchymal-like state by upregulating mesenchymal markers and downregulating epithelial markers; and this phenotype was reversed upon CTBP1 knockdown (Figure 6I). Overall, all these data confirm that CTBP1 is a major functional target of miR-644a mediating drug resistance and EMT in breast cancer.

### miR-644a/CTBP1-mediated wild type or mutant p53 upregulation acts as a switch deciding on G1 arrest or apoptosis

**Figure 7:**
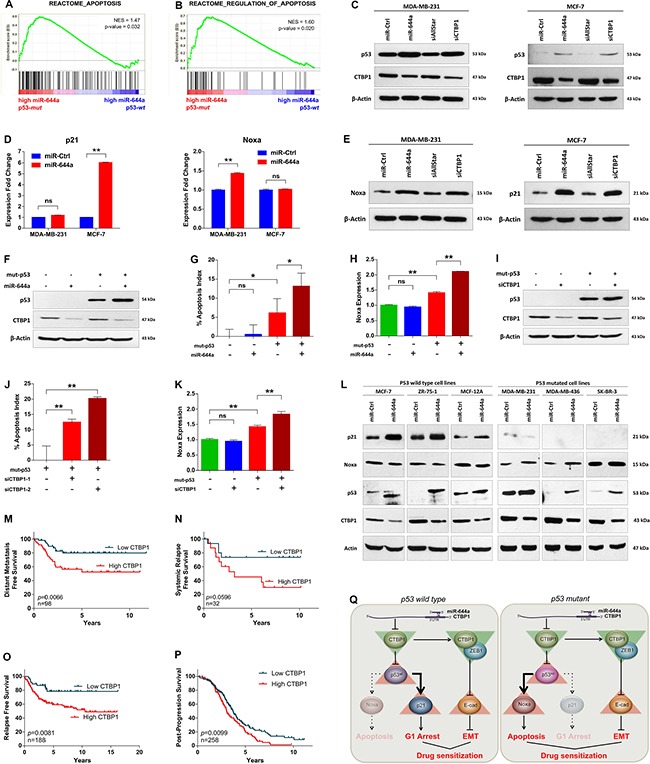
miR-644a/CTBP1-mediated wild type or mutant p53 upregulation acts as a switch on G1-arrest or apoptosis, and CTBP1 expression predicts survival of patients with p53 mutation (**A** and **B**) Enrichment plots of patients from GSE22220 with high miR-644a levels (*n* = 105). Among patients with high miR-644a, genes annotated to Apoptosis (A) and Regulation of Apoptosis (B) pathways in Reactome were significantly enriched in p53-*mut* group as compared to p53-*wt* group. (**C**) Western Blot analysis showing the regulation of p53 in MDA-MB-231 (left) and MCF-7 cells (right) upon miR-644a overexpression or CTBP1 knockdown. (**D** and **E**) qRT-PCR (D) and western blot (E) analysis of p21 and Noxa gene expression in MDA-MB-231 and MCF-7 cells upon miR-644a overexpression or CTBP1 knockdown. (**F**–**K**) Changes in the apoptotic index based on Caspase-3 cleavage in p53-*wt* MCF-7 cells transfected with miR-644a (G) or siCTBP1–1, siCTBP1–2 (J) together with *mut*-p53 ORF. Regulation of Noxa expression upon miR-644a overexpression (H) or CTBP1 knockdown (K) in the presence of *mut*-p53 was shown with qRT-PCR analysis. Overexpression of *mut*-p53 in p53-*wt* MCF-7 cells was confirmed with Western Blot analysis (F and I). (**L**) Western blot analysis showing CTBP1, p53, Noxa and p21 expression upon miR-644a upregulation using mimics in 6 different breast cancer cell lines. (**M** and **N**) Kaplan Meier survival curves of breast cancer patients with p53 mutation based on CTBP1 median expression levels in datasets GSE58644 (*n* = 98) representing percentage distant metastasis-free survival (M) and in GSE19536 (*n* = 32) representing percentage systemic relapse-free survival (N). (**O** and **P**) Kaplan Meier survival curve of breast (O) and ovarian (P) cancer patients with p53 mutation based on ‘best cut-off’ for CTBP1 expression levels in KM Plotter (*n* = 188 for O, *n* = 258 for P) representing percentage relapse-free (O) and post-progression (P) survival. (**Q**) Schematic description of miR-644a/CTBP1/p53 axis-mediated drug resistance by simultaneous modulation of cell survival and EMT in p53-*wt* (left) and p53-*mut* (right) cells.

The *in vitro* findings that overexpression of miR-644a or loss of its target CTBP1 induces apoptosis in p53-*mut*, but not in p53-*wt* cells triggered us to investigate the relationship between miR-644a, CTBP1, and p53 mutation status. First, we performed an enrichment analysis for apoptosis-related gene sets on p53-*mut* vs p53-*wt* patients that are expressing high levels of miR-644a from GSE22220 dataset [31]. We have separated the patients as p53-*wt* and p53-*mut* according to the expression levels of a gene signature associated with p53 status [32] (details are given in Material and Methods section and Supplementary Figures S8 and S9). We observed that apoptosis genes were significantly enriched in p53-*mu*t patients compared to p53-*wt* patients (Figure 7A and 7B). While investigating the underlying mechanism, we found that miR-644a overexpression or CTBP1 knockdown increased the expression of p53 level in both p53-*mut* MDA-MB-231 and p53-*wt* MCF-7 cells (Figure 7C). Furthermore, in 231.miR-644a cells, p53 level was reduced upon CTBP1 ORF expression, which shows that miR-644a mediated upregulation of p53 is *via* CTBP1 downregulation (Supplementary Figure S6A). Notably, overexpression of miR-644a or knockdown of CTBP1 in MCF-7 cells did not change p53 mRNA level, which indicates a possible post-transcriptional regulation of p53 expression (Supplementary Figure S6B and S6C). Overall, these results show that miR-644a induces p53 expression/activity in a p53 status-independent manner.

It has been shown that stabilized p53 activates several genes that induce cell cycle arrest (e.g. p21) and apoptosis (e.g. Noxa, Bax and Puma) [33]. Furthermore, CTBP1 knock-out cells were shown to express high levels of pro-apoptotic genes Noxa and Bax [34]. Therefore, we examined the expression of these in both MDA-MB-231 and MCF-7 cells upon miR-644a overexpression. While the expression of p21 was only upregulated in p53-*wt* MCF-7 cells, Noxa, but neither Bax nor Puma, was significantly upregulated in p53-*mut* MDA-MB-231 cells at mRNA and protein level upon miR-644a transfection (Figure 7D and 7E; Supplementary Figure S6D and S6E). Analysis of commonly upregulated genes by *wt*-p53 in breast cancer patients from Troester *et al.* [32] and loss of CTBP1 in MCF-7 cells from Di et *al.* [35] also identified only p21 and BTG2 (data not shown) which further supported that miR-644a mediated upregulation of *wt*-p53 and p21 is *via* CTBP1 downregulation.

We then asked whether we can induce apoptosis upon miR-644a expression or CTBP1 knockdown in p53-*wt* MCF-7 cells if *mut*-p53 was co-expressed. After confirming successful overexpression of *mut*-p53 with western blot (Figure 7F and 7I), we performed an apoptosis assay followed by qRT-PCR of Noxa. Overexpression of *mut*-p53 alone in p53-*wt* MCF-7 cells induced a certain level of apoptosis and Noxa expression, which is further enhanced either by overexpression of miR-644a or by knockdown of CTBP1 (Figure 7G–7K). This confirms that miR-644a mediates apoptosis in the presence of *mut*-p53 mainly by the pro-apoptotic gene Noxa. Furthermore, presence of *mut*-p53 did not affect downregulation of CTBP1 (Figure 7F and 7I; Supplementary Figure S6F and S6H) suggesting that there is no feedback regulation of CTBP1 by p53 in the miR-644a/CTBP1/p53 axis. Lastly, to validate that p53 serves as a switch in miR-644a/CTBP1/p53 axis in breast cancer, we checked the downstream effects of miR-644a in 6 different breast cancer cell lines (three p53-*wt* and three p53-*mut*) after forced miR-644a expression using mimics. We demonstrated that depending on the p53 status, miR-644a/CTBP1/p53 axis leads to either p21 upregulation (in case of p53-*wt* cells) or Noxa upregulation (in case of p53-*mut* cells) explaining the observed G1 arrest or apoptosis induction, respectively (Figure 7L).

### p53 mutant patients with high CTBP1 level are predicted to have a worse survival

It has been known that p53-*wt* patients have better survival as compared to p53-*mut* ones [36–38]. We confirmed this by analyzing published patient data and showed that p53-*mut* patients survive less than p53-*wt* patients (Supplementary Figure S7A and 7B). Unlike the prognostic relevance of miR-644a signature, no correlation of CTBP1 mRNA levels with the survival of breast cancer patients from GSE58644 and GSE19536 [39] datasets was found (Supplementary Figure S7C and S7D). Therefore, we asked if the expression level of CTBP1 can be associated with the survival of p53-*mut* patients in these datasets. This was indeed the case for both of the datasets as well as an online survival analysis tool, Kaplan-Meier (KM) Plotter [40] (Figure 7M–7O). However, in p53-*wt* patients CTBP1 expression level did not have any significant effect on survival of the patients (Supplementary Figure S7E–S7G). We observed a similar pattern in ovarian cancer where p53-*mut* patients with high CTBP1 levels are less likely to survive compared to patients with low CTBP1 (Figure 7P; Supplementary Figure S7H). Overall, these data suggest that CTBP1 expression may be associated with survival of p53-*mut* breast and ovarian cancer patients.

## DISCUSSION

Little is known about the deregulation of miR-644a in cancer. It has been shown that high expression of miR-644a (previously known as miR-644 according to miRBase release 21 [41]) is correlated with shorter overall survival in Acute myeloid leukemia (AML) [42], and miR-644a is found to be upregulated in bladder cancer [43]. On the contrary, miR-644a overexpression was shown to downregulate an isoform of the androgen receptor, and decrease viability in prostate cancer cell lines [44]. However, there is no functional study on miR-644a, and its deregulation in breast cancer has not been reported. Here, we demonstrated that miR-644a acts as a tumor suppressor in breast cancer by regulating tumor progression, metastasis, and drug resistance affecting the patient survival (Figure 7Q). We identified the transcriptional co-repressor CTBP1 as the major functional target of miR-644a phenocopying all its effects on cell proliferation, apoptosis, EMT and drug resistance. Its loss upon miR-644a overexpression on one hand leads to G1 arrest or apoptosis by increasing p53 expression and on the other hand leads to increased E-Cadherin to inhibit EMT and metastasis.

It has been known that activated p53 can either lead to apoptosis or cell cycle arrest in a highly context dependent manner. As reviewed by Haupt *et al.* [45] and Fridman *et al.* [46], cell type, strength and nature of the stimulus as well as the presence of collateral signals can determine cell fate in the presence of a stress stimulus. Importance of the latter has been shown in case of DNA damage during which Myc shifts the balance of cell fate from cell cycle arrest to apoptosis by blocking p21 induction *via* recruiting Miz-1 to p21 promoter site, and thereby preventing p53-mediated transcription [47, 48]. Therefore, we also checked changes in Myc expression with miR-644a overexpression, but did not observe an induction in Myc levels (data not shown), which might be due to the presence of a stimulus other than DNA damage in our system. Here, we propose that the mutation status of p53 is yet another factor that is important for the decision of undergoing either to cell cycle arrest or to apoptosis. Our results demonstrated that increase in p53 levels upon miR-644a overexpression or CTBP1 knockdown increases p21 which protects cells from p53-dependent apoptosis [17], and causes cell cycle arrest in p53-wt cells whereas it induces apoptosis *via* increasing the expression of pro-apoptotic gene Noxa in p53-*mut* cells. We saw that overexpression of *mut*-p53 in p53-*wt* MCF-7 cells was enough to shift the balance between cell cycle arrest and apoptosis in favor of apoptosis through upregulation of an established pro-apoptotic BH3-only protein, Noxa, even though p21 was still induced (Supplementary Figure S6G and S6I).

p53 is mutated in 30% of breast cancer which causes several defects in p53 functioning like altered DNA binding affinity or loss of transcriptional activity [49, 50]. However, there is substantial evidence showing that mutant p53 is still able to induce apoptosis through different mechanisms. It has been shown that a transcriptionally inactive mutant p53 can still activate the pro-apoptotic gene Bax upon DNA damage [51], and some transcription-defective mutants retain significant apoptotic activity independent of Bax induction [52, 53]. These suggest that in case of p53 mutation, a “gain of pathway” phenomenon occurs which may involve either transcription-dependent or independent activation of a different set of pro-apoptotic genes [54]. Our results support these findings with regard to induction of apoptosis by miR-644a in p53-mutant breast cancer cells by activation of Noxa. Notably, although we found no correlation of CTBP1 levels with the survival of breast cancer patients (Supplementary Figure S7C–S7H), in all datasets that we analyzed, we confirm that p53-*mut* patients with breast or ovarian cancer that show high CTBP1 level are associated with a worse survival as compared to the patients with low CTBP1 group (Figure 7M–7P). This suggests that CTBP1 could be a potential prognostic factor for breast cancer patients with p53 mutations which may be due to the fact that apoptosis induction is more effective on prolonging overall survival of patients than p53-dependent growth arrest.

As EMT and cell survival are closely related with drug resistance, we examined the effects of miR-644a and its target CTBP1 on drug sensitization, and observed that overexpression of miR-644a sensitized different breast cancer cells representing different subtypes to both chemotherapy and targeted therapy agents e.g. tamoxifen and gefitinib. In addition, low CTBP1 correlated with better response (Figure 6A) and longer distant metastasis-free survival of breast cancer patients treated with chemotherapy (Figure 6B). We have previously shown that miR-375 blocks EMT and sensitizes MCF-7 cells to tamoxifen [55]. Similarly, it has been shown that miR-147 blocks EMT and sensitizes colon cancer cells to gefinitib [56]. Although these drugs have different targets and mechanisms of action, we reveal miR-644a as a pleotropic sensitizer, which suggests that the inhibition of EMT might be a common nominator for sensitization to all drugs tested. However, as CTBP1 can increase the expression of MDR1 gene transcriptionally [57], we cannot rule out the alternative of possible downregulation of MDR1 upon miR-644a expression, which leads to inhibition of multi-drug resistance. Nevertheless, our results suggest that miR-644a or its target CTBP1 could be a potential drug candidate which can simultaneously block primary tumor growth, metastasis, and finally sensitize cancer cells to several different drugs. A recent study reported a small molecule, NSC95397, which inhibits the interaction between CTBP1 and its binding partners and blocks the CTBP1-mediated transcriptional repression [58]. Although using miRNAs as potential drugs could need longer time, small molecules targeting CTBP1 could act as potential drugs for cancer therapy in the near future. In conclusion, the miR-644a/CTBP1/p53 axis acts not only as biomarker of progression and drug response, but also could be targeted for cancer therapy.

## MATERIALS AND METHODS

### Cell culture and reagents

Human breast cancer cell lines MDA-MB-231, MCF-7, BT474, SK-BR-3, ZR-75–1 together with normal breast epithelial cell lines MCF-10A and MCF-12A were obtained from ATCC (Manassas, VA, USA). MDA-MB-231 and SK-BR-3 cell lines were cultured with Dulbecco Modified Eagle Medium (Lonza, NJ, USA) while MCF-7, BT474 and ZR-75–1 cell lines were cultured with DMEM supplemented with 0.1% insulin. MCF10A and MCF-12A cell line was cultured with DMEM (Lonza, NJ, USA) supplemented with 0.1% insulin (0.01 mg/ml), 0.002% EGF (20 ng/ml) (both from Sigma-Aldrich, Saint-Louis, MO, USA). All media were supplemented with 50 U/ml penicillin/streptomycin, 1% non-essential amino acids and 10% fetal bovine serum (Lonza, NJ, USA). All cell lines were tested for mycoplasma contamination regularly using MycoAlert mycoplasma detection kit (Lonza, NJ, USA).

### Transient transfection with miRNA mimics, hairpin inhibitors, siRNAs and expression constructs

Transfections were carried out as previously described [59]. Sequence of miR-644a mimic was AGUGUGGCUUUCUUAGAGC. For miRNA mimic viability screen, miRNA mimics were transfected at a concentration of 20 nM for 48 hours. For other experiments, miR-644a and all siRNA transfections were done at a concentration of 40 nM whereas hairpin inhibitors were transfected at a concentration of 100 nM for either 48 hours or 72 hours. 50 ng (for 96-well experiments) or 500 ng (for 6-well experiments) per well of GFP tagged CTBP1 (NM_001012614; Cat. No. RG208594) human ORF Clone and Myc-DDK tagged TP53 (NM_000546; Cat. No. RC200003) human mutant ORF Clone vector were purchased from Origene and used for overexpression and rescue experiments. Latter one expresses transcript variant 1 of Homo sapiens protein p53 having an R175H mutation.

### Plasmid construction and site-directed mutagenesis

The construction of plasmid carrying 3′-UTR sequence of CTBP1 gene was done as previously described [9]. The 967 bp length 3′-UTR which contained last exon sequence common to two transcript variants of CTBP1 and annotated CCDS in NCBI Consensus Coding Sequence database [60] was used to design the primers. Primers used were 5′-GGCACTCGAGCTGCTGTGGAAGGTAT-3′ and 5′-ATACAAGCGGCCGCAGTCACAAACATGATTTTA AC-3′. For site-directed mutagenesis, the predicted hsa-miR-644a target site of the previously described psiCheck2/CTBP1–3′UTRwt construct were disrupted by four point mutations in the seed region (Supplementary Figure S3A).

### Quantitative RT-PCR analysis of mRNAs and miRNAs

Total RNA was isolated using TRIsure (Bioline, Luckenwalde, Germany). cDNA synthesis was performed using RevertAid RT Reverse Transcription Kit (Life Technologies), following manufacturer's instructions. Each real-time PCR assay was carried out in triplicates using LightCycler 480 SYBR Green I Master kit (Roche). Sequences of the primers were provided elsewhere (Supplementary Table S5). ACTB and HPRT were used as housekeeping genes. Data were analyzed according to ΔΔ*C*_T_ method [61]. Quantitative RT-PCR analysis for miRNAs was done as previously described [9]. RNU44 was used as the housekeeping gene.

### Western blot

Whole-cell lysates were prepared using RIPA lysis buffer containing protease inhibitor Complete Mini (Roche, Basel), anti-phosphatase PhosSTOP (Roche, Basel), 10 mM NaF and 1 mM Na4VO3. Protein concentrations were determined with BCA Protein Assay Reagent Kit (Thermo Scientific, Rockford, IL). Proteins were denatured with 4× loading dye containing 10% SDS and 50% glycerol at 95°C for 5 minutes, and 20 μg proteins were loaded in each lane. Protein samples were separated by SDS-PAGE and incubated with primary antibodies (Supplementary Table S6). Horseradish peroxidase conjugated anti-mouse or anti-rabbit antibodies (Cell signaling Technology, USA) were used as secondary antibodies, and signals were detected by enhanced chemiluminescence (Thermo Scientific, Rockford, IL).

### Cell cycle, viability and apoptosis assays

Cell cycle analysis was performed as previously described [62]. Transfections were done in 6-well plates (2.5 × 10^5^ cells per well) in triplicates. For drug sensitization assays, drug treatments were done one day after miRNA mimic or siRNA transfections with doxorubicin (0.05–10 uM), cisplatin (0.001–30 uM), tamoxifen (2.5–40 uM) or gefitinib (0.1–20 uM) for 72 hours. Cell viability (including viability of miRNA mimic screen and drug sensitization assays) and apoptosis were assessed by Cell Titer Glo Luminescent Cell Viability and Caspase-Glo 3/7 assays (Promega, WI, USA), respectively according to the manufacturer's instructions. Cell viability after polyHEMA assay was assessed with WST-1 assay (Roche) according to the manufacturer's instructions.

### RTCA proliferation and migration assays

Real time growth and migration of MDA-MB-231 and MDA-MB-436 cells were monitored using xCelligence Real-time Cell Analyzer system (Acea Biosciences, San Diego, CA, USA) as previously described [9, 59]. For invasion assay, cells were transfected as described above and seeded in Matrigel invasion chambers (BD Pharmingen) in 1% FBS DMEM. After 48 hours, number of invaded cells were counted by flow cytometry (FACS Calibur) and analyzed by Cell Quest Pro software (BD Bioscience). In wound healing assay, 1 × 10^5^ cells were seeded on 24-well plates, and pictures were taken with Axiovert 25 light microscope (Hund, Wetzlar, Germany). PolyHEMA assay was performed as previously described [55].

### Dual luciferase reporter assay

Dual-luciferase reporter assay was performed as previously described [9]. Luciferase activity was measured in Synergy HT microplate reader machine (BioTek, Vermont, US) after 24 hours of transfection, and values were normalized to firefly luciferase activity.

### Immunofluorescence staining and microscopy

Nuclear staining of cells were done with 4, 6-diamidino-2-phenylindole (DAPI) and filamentous actin were stained with Alexa Fluor 488 phalloidin (1:40; Invitrogen) as previously described [9]. Coverslips were mounted with Shandon Immu-Mount reagent. Images were taken using a Zeiss microscopy.

### Lentiviral vector constructs and infection

SMARTchoice human lentiviral hsa-miR-644a shMIMIC hCMV-turboGFP, GIPZ human CTBP1 shRNA vectors with clone IDs V3LHS_380132, V3LHS_398420 and V3LHS_113279 encoding different shRNAs sequences and GIPZ non-silencing lentiviral shRNA control were purchased from Dharmacon (Lafayette, CO). MDA-MB-231.luc cells (a kind gift from Dr. Dihua Yu (MD Anderson, TX)) were transduced with miR-644a viral particles in 24-wells plate, and 96 hours post-transduction, selection with 1 μg/ml of puromycin was started. To produce viral particles with CTBP1 shRNAs and non-silencing shRNA control, 6 μg of each of these vectors and 4.3 μl of trans-lentiviral packaging mix (Dharmacon) were used to co-transfect HEK293FT cells in 6-wells plate with CaCl_2_ reagent (Dharmacon). 48 hours post-transfection, first viral particles were harvested. 96 hours post-transduction, selection with 1 μg/ml of puromycin was started. LentiCRISPRv2 vector was a kind gift from Dr. Feng Zhang (MIT, Boston, MA) [63]. For sgRNA design (Supplementary Figure S3B and S3C), candidate target sequences were determined using E-CRISPR tool [64]. For the packaging of pLentiCRISPR/CTBP1 sgRNA1 and pLentiCRISPR/CTBP1 sgRNA2 vectors, 30% confluent HEK293FT cells in 100 mm plates have been co-transfected using 42 μg of these vectors, 31.5 μg of pMD2.G (Addgene) and 21 μg psPAX2 (Addgene). Transfection, transduction and selection were performed as described previously for GIPZ vectors series.

### *In vivo* experiments

All animal experiments have been approved by the animal ethics committee of Bilkent University. For primary tumors, 6–8 week old female athymic Nu/nu mice were injected with 2 × 10^6^ cells into both left and right mammary fat pads (MFP) subcutaneously without incision, with 5–6 mice per group. Tumor growth was monitored by measuring the tumor volume twice a week with a caliper after tumors become palpable. Tumor volumes were calculated as length × width^2^/2. All the mice were sacrificed once one of the mice reached threshold of 1500 mm^3^ volume, and tumors were collected and weighed. For doxorubicin response experiments, primary tumors were developed using above mentioned protocol, and mice were treated with a dose of 5 mg/kg body weight of mice weekly through intravenous injection when tumors became palpable. In the beginning, all tumors showed sensitivity towards treatment and showed decrease in volume. Some of these sensitive tumors were collected after 8–10 weeks of treatment. Later, remaining tumors started to increase in size, and showed resistance towards treatment, and these resistant tumors were collected after 12–15 weeks of treatment. For tail-vein metastasis assay, 1.5 × 10^6^ cells were injected into tail vein of each 6–8 weeks old female athymic Nu/nu mice, with 3–5 mice per group. The mice were sacrificed, and lung metastasis was evaluated once one of the mice became moribund with a luciferase assay. Due to heterogeneous distribution of nodules, three different parts were randomly collected from each lung to make a tissue pool and weighed for normalization. Lung tissues were ground in cold PBS by tissue homogenizer and treated with lysis buffer (Promega). 500 ul lysis buffer was added to 140–180 mg weight. After 15 min incubation, 100 ul of luciferase substrate (Promega) was added to 20 ul of the lysed sample, and luminescence was measured with a luminometer. For Bouin's fixation, harvested lungs were cleaned with PBS and placed into Bouin's solution on a shaker for overnight. After fixation they were kept in 70% alcohol. The lung samples used for Bouin's fixation were excluded from luciferase assay and H&E staining.

### miRNA target prediction

Targetscan release 6.2 (http://www.targetscan.org), PITA miRNA target recognition from Segal Lab of Computational Biology (http://genie.weizmann.ac.il) and miRDB (http://mirdb.org/miRDB/index.html) was used as target prediction algorithms for identification of miR-644a targets. Common predicted targets between all three databases and downregulated genes by miR-644a (microarray results) were represented in a Venn diagram using Venny 2.0.

### Microarray analysis

Expression profiling data were normalized with quantile normalization. Quality control and differential gene expression analysis was conducted using *limma* in Bioconductor [65]. Microarray data can be retrieved from NCBI Gene Expression Omnibus (GEO) database with the accession number GSE82058.

### Patient data and statistical analysis

Cell line and patient data were retrieved from the NCBI GEO database (GSE4922, GSE2603, GSE16446, GSE19536, GSE22220, GSE38167, GSE40059, GSE45666, GSE58606 and GSE58644) and from the Molecular Taxonomy of Breast Cancer International Consortium (METABRIC) project with data deposited in EMBL European Genome–Phenome Archive (http://www.ebi.ac.uk/ega/), with an accession number EGAS00000000122. A brief summary of these datasets is provided in Supplementary Table S4. For GSE19536, patient data were retrieved from GSE3985 which is a former study done with the same patient samples and which is providing more detailed patient data. Significance scores of genes up and down-regulated by miR-644a were calculated as previously described [66]. miR-644a signature score was defined as the ratio of the significance scores of genes up and down-regulated by miR-644a. For the GSEA analysis, patients in GSE58644 dataset were grouped based on their miR-644a significance score ratios, which we defined as the miR-644a signature score. For the GSEA analysis with GSE22220 dataset, patients expressing high levels of miR-644a were separated into two groups as p53-*mut* and p53-*wt*. Survival curves were generated using Kaplan-Meier method. Patients without any available survival time or event were excluded from the corresponding patient groups. All separations were done from median. For KM Plotter curves, ‘best cut-off’ option was selected for the separation of patients. Significance of the differences in survival between two groups was calculated by Log-rank (Mantel-Cox) test. For the separation of patients as p53-*wt* or p53-*mut*, data provided by the publishers of the studies were utilized if available. In GSE58644 and GSE22220, the separation was done by clustering patients according to their expression levels of a gene signature associated with p53 status [32]. The accuracy of p53 status prediction with this gene signature was tested by using data from GSE19536 in which p53 status of the patients was provided. 85% and 77% of the p53-*mut* and p53-*wt* patients, respectively could be predicted from their expression levels of the p53 status signature. The generated heatmap is provided as Supplementary Figure S8. Heatmaps for the other datasets with accession numbers GSE58644 and GSE22220 are provided as Supplementary Figure S9. Gene ontology analyses were done with DAVID bioinformatics tool [67]. For the microarray datasets that contain negative expression values, a small number was added such that all values became positive [68]. Comparisons between two groups were made by two-tailed student *t*-test. Significance cut-off were taken as *P* = 0.05. For the real-time cell growth experiments, data were normalized to time of transfection and statistical significance was determined with a paired, two-tailed student *t*-test. Statistical significance between two dose response curves was also determined with a paired, two-tailed student *t*-test.

## SUPPLEMENTARY MATERIALS FIGURES AND TABLES




