# Supplementary material for: The miR-644a/CTBP1/p53 axis suppresses drug resistance by simultaneous inhibition of cell survival and epithelial-mesenchymal transition in breast cancer
Source: Oncotarget. 2016 Jul 8;7(31):49859–77. doi: 10.18632/oncotarget.10489 (PMC5226553; doi:10.18632/oncotarget.10489)
Supplement: Supplementary file 1 [file oncotarget-07-49859-s001.pdf]

The miR-644a/CTBP1/p53 axis suppresses drug resistance by simultaneous inhibition of cell survival and epithelial-mesenchymal transition in breast cancer

Supplementary Materials

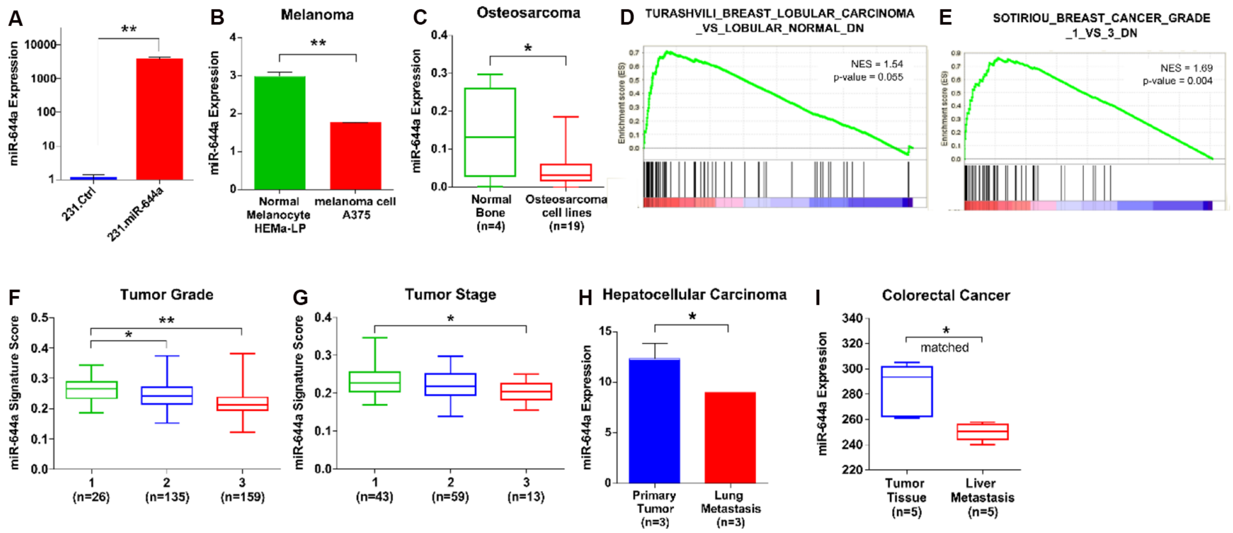

Supplementary Figure S1: Confirmation of miR-644a overexpression in 231.miR-644a stable cells and correlation of miR-644a loss with formation and metastasis of different cancers. (**A**) qRT-PCR analysis of miR-644a in 231.miR-644a stable cells. (**B** and **C**) miR-644a expression in melanoma (B) and osteosarcoma cell lines (C) as compared to a normal melanocyte cell line and normal bone, respectively. (**D** and **E**) Enrichment plots of patients from GSE58644 (n = 320) with high or low miR-644a signature score. Genes downregulated in breast lobular carcinoma as compared to normal lobular breast tissues (D) were enriched in patients expressing high levels of miR-644a signature score. Genes downregulated as breast tumors progress through histologic grade 3 were enriched in patients with high miR-644a signature score (E). (**F** and **G**) Changes in miR-644a signature score with tumor grade (F) and stage (G) in patients from GSE58644. (**H** and **I**) miR-644a expression in a lung metastasis of hepatocellular carcinoma (H) and liver metastasis of colorectal cancer (I) as compared to primary tumors. Statistical significance was indicated (*p < 0.05; **p < 0.01; ns, not significant). Column data represent mean ± SD. Box-plots depict median number and the 25th to 75th quartiles. Upper and lower whiskers represent the minimum and maximum values in the corresponding group. This applies to all figures shown.

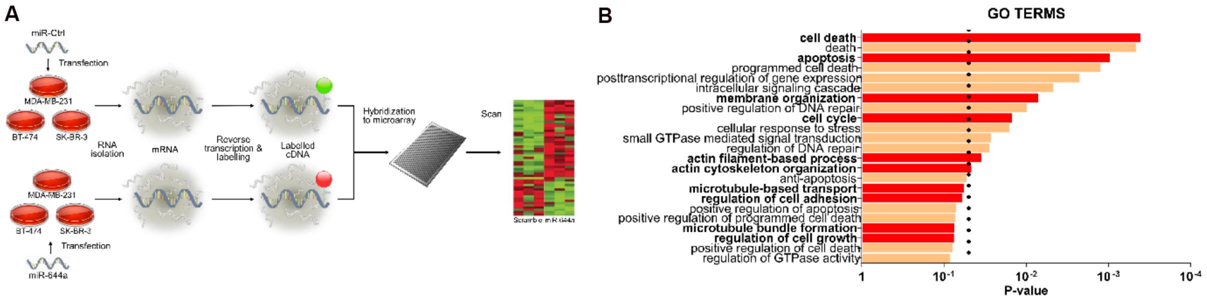

Supplementary Figure S2: Identification of miR-644a gene signature and its roles in cancer. (**A**) Scheme for microarray analysis performed to obtain a miR-644a gene signature. (**B**) GO terms associated with miR-644a signature.

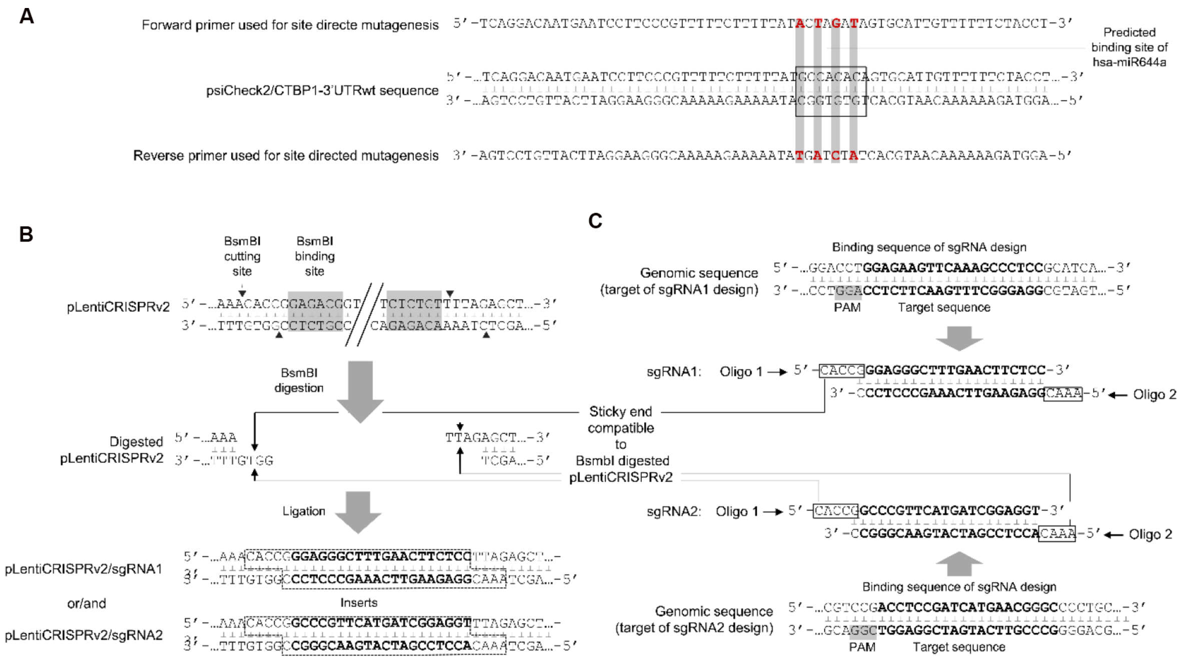

Supplementary Figure S3: Primer and sgRNA design for site-directed mutagenesis and CTBP1 knockout. (**A**) Primer sequences used for site-directed mutagenesis assay. (**B** and **C**) Design of sgRNAs used in CRISPR-Cas9 mediated knock-out of CTBP1.

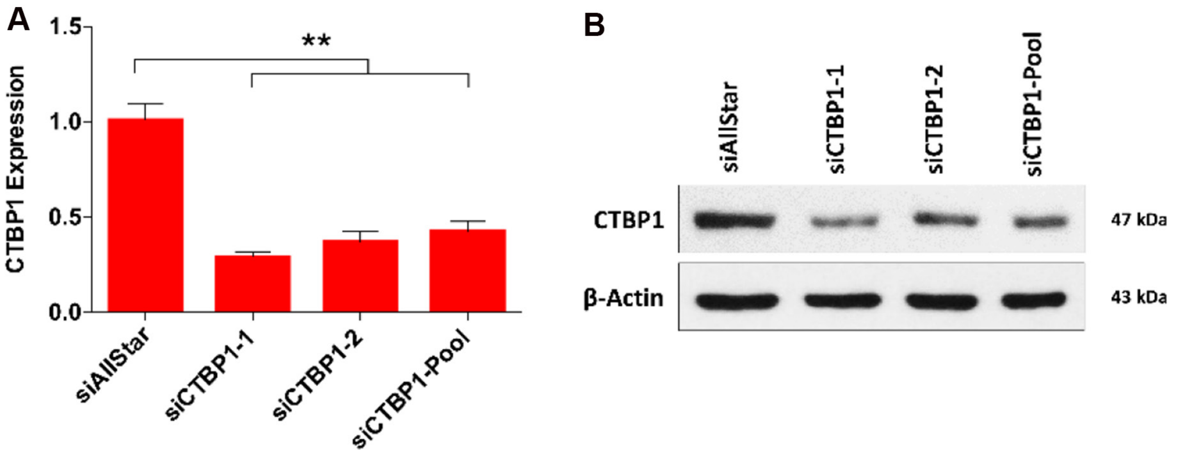

Supplementary Figure S4: Confirmation of CTBP1 knockdown with siRNA silencing. (**A** and **B**) qRT-PCR (A) and Western Blot (B) analysis of CTBP1 levels in MDA-MB-231 cells transfected with control siRNA (siAllstar) or different CTBP1 targeting siRNAs (siCTBP1-1, siCTBP1-2 and siCTBP1-Pool). *n* = 3 for A.

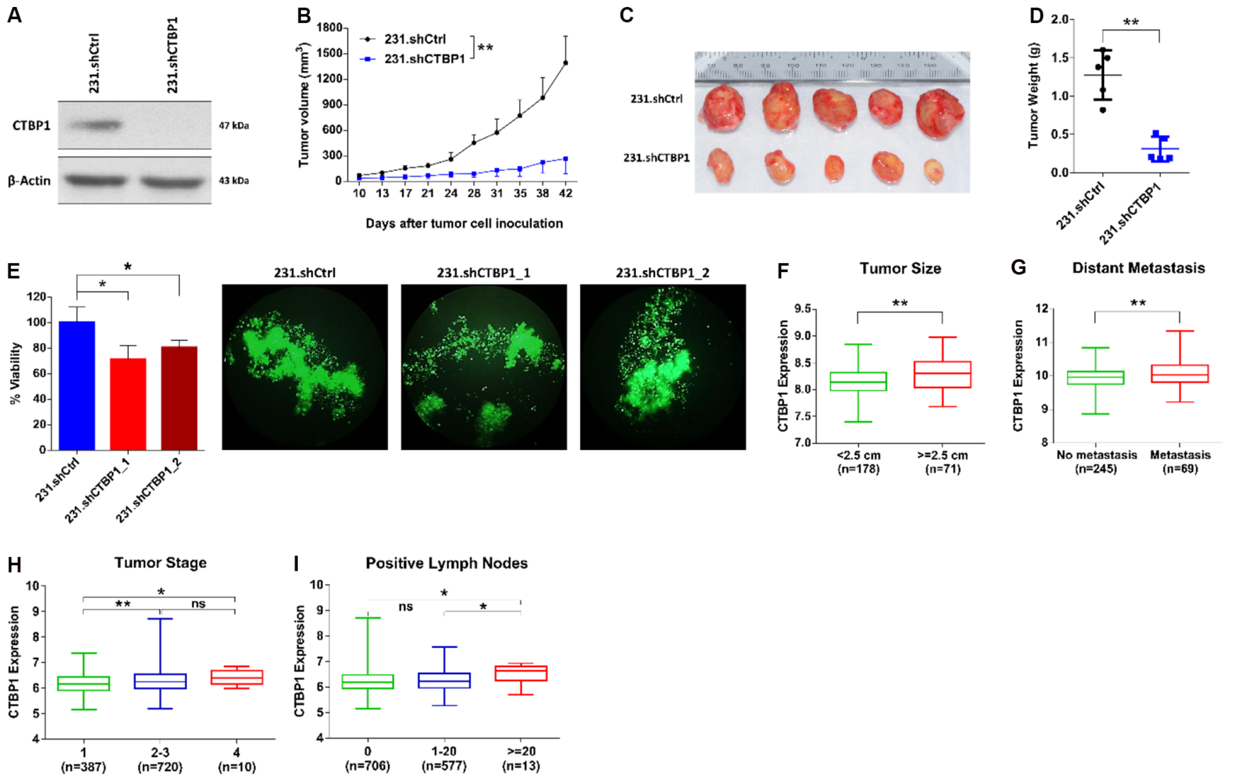

Supplementary Figure S5: CTBP1 is mediating breast cancer formation, progression and metastasis. (**A**) Western Blot analysis of CTBP1 levels in MDA-MD-231 cells stably expressing either a non-targeting shRNA (231.shCtrl) or a CTBP1 targeting shRNA (231.shCTBP1) confirming stable knockdown of CTBP1. (**B**) Tumor progression in xenografts generated with orthotopic injection of 231.shCtrl or 231.shCTBP1 cells. *n* = 5. (**C**) Representative images of tumors collected from xenografts of (B) on day 42. (**D**) Tumor weights in xenografts from (B) at day 42. (**E**)Viability of 231.shCtrl, 231.shCTBP1_1 and shCTBP1_2 cells grown in anchorage-independent conditions for 7 days, quantified by WST-1 assay (left) together with their fluorescence microscopy images with 10X magnification (right). (**F** and **G**) Changes in CTBP1 expression in breast tumors with tumor size from GSE4922 (F) and with presence of distant metastasis (G) from GSE58644. (**H** and **I**) Changes in CTBP1 expression in breast tumors with tumor stage (H) and number of positive lymph nodes (I) from METABRIC data.

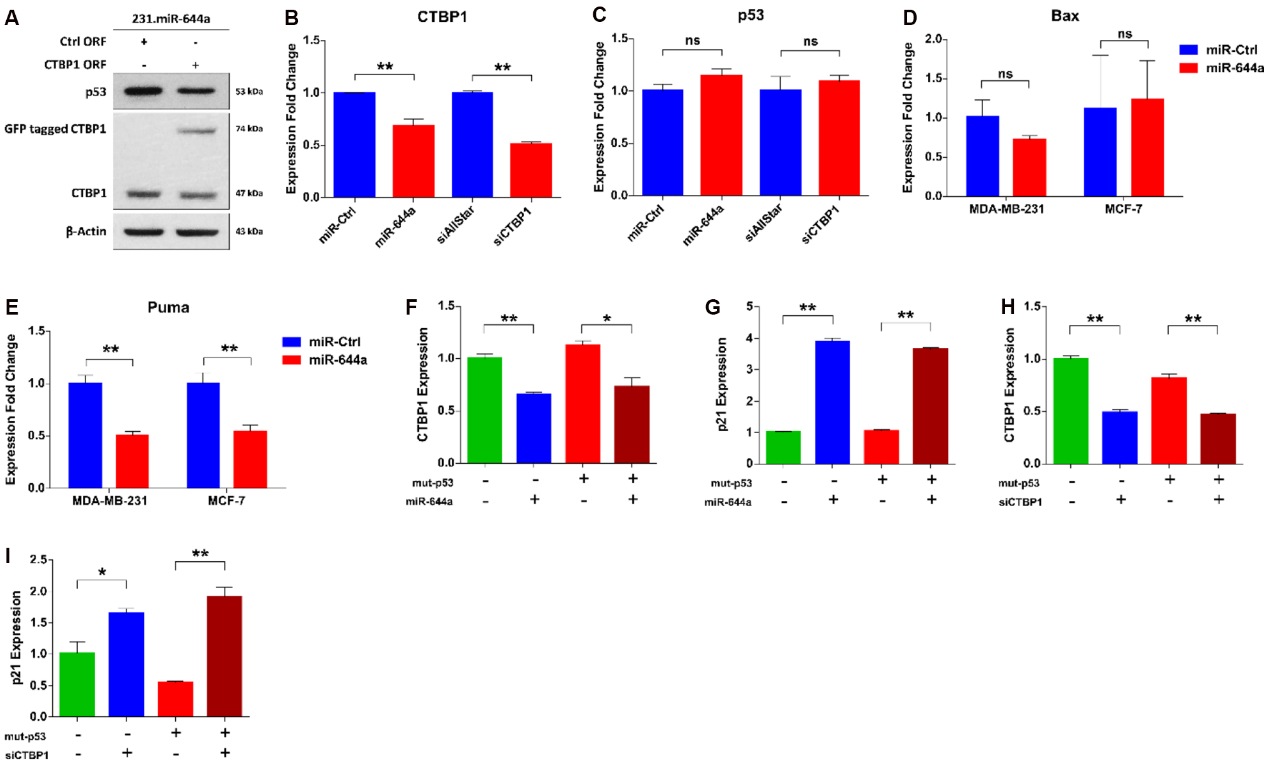

Supplementary Figure S6: Effects of miR-644a or CTBP1 knockdown on p53, p21 and two apoptotic markers, Bax and Puma expression in the absence or presence of mut-p53. (**A**) Western Blot analysis of p53 and CTBP1 levels in 231.miR-644a cells transfected with either Ctrl ORF or CTBP1 ORF showing downregulation of p53 with rescue of CTBP1 expression. (**B** and **C**) qRT-PCR analysis of CTBP1 (B) and p53 (C) expression in MCF-7 cells upon miR-644a overexpression or CTBP1 knockdown with siCTBP1-Pool. (**D** and **E**) qRT-PCR analysis of Bax (D) and Puma (E) expression in MDA-MB-231 and MCF-7 cells transfected with miR-Ctrl or miR-644a. (**F**–**I**) qRT-PCR analysis of CTBP1 (F and H) and p21 (G and I) expression in p53-*wt* MCF-7 cells overexpressing miR-644a or having knockdown of CTBP1 in the absence or presence of *mut*-p53.

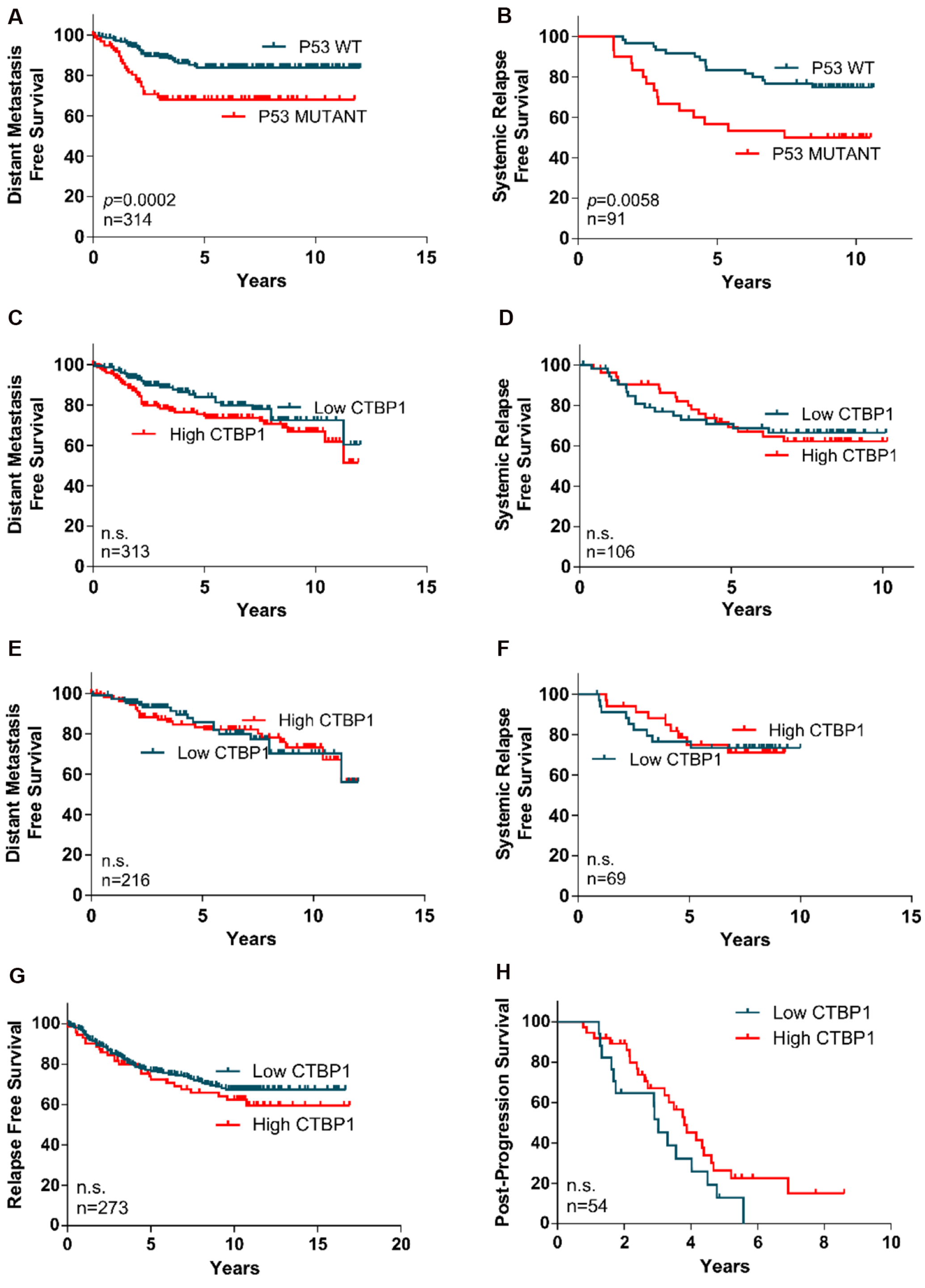

Supplementary Figure S7: Effects of p53 status and CTBP1 levels on the survival of breast and ovarian cancer patients. (**A** and **B**) Kaplan Meier survival curves of breast cancer patients based on p53 status in datasets GSE58644 representing percentage distant metastasis free survival (A) and in GSE19536 representing percentage systemic relapse free survival (B). (**C** and **D**) Kaplan Meier survival curves of breast cancer patients based on CTBP1 median expression from datasets in A and B. (**E** and **F**) Kaplan Meier survival curves of p53-*wt* breast cancer patients based on CTBP1 median expression from datasets in A and B. (**G** and **H**) Kaplan Meier survival curve of p53-*wt* breast (G) and ovarian (H) cancer patients based on best cut-off for CTBP1 expression levels in KM Plotter (n = 273 for G, n = 54 for H) representing percentage relapse free (G) and post-progression (H) survival.

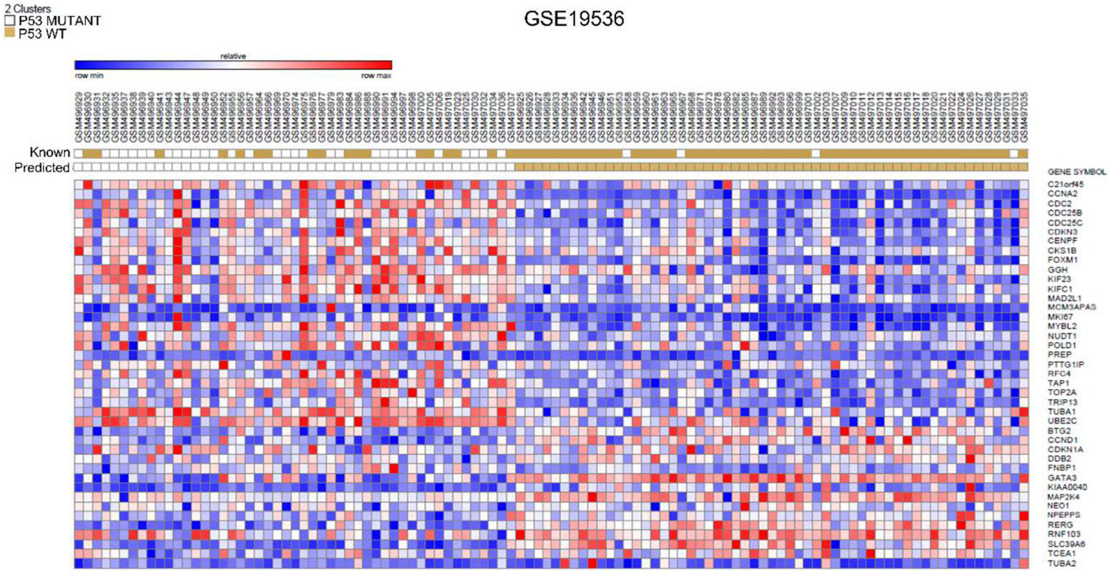

Supplementary Figure S8: Validation of the p53 status signature in a dataset containing patients with known p53 status. Heatmap of patients from GSE19536 clustered as p53-*wt* and p53-*mut* according to p53 status signature expression together with their known p53 status.

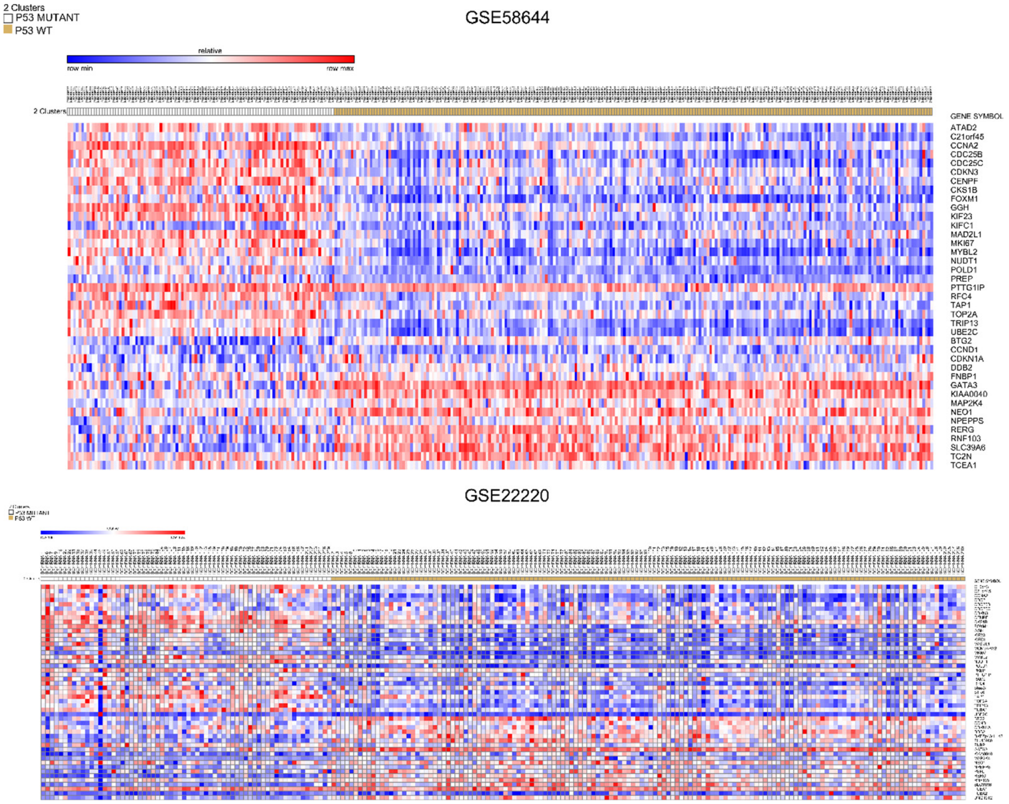

Supplementary Figure S9: Separation of patients in two different datasets according to p53 status. Heatmap of patients from GSE58644 and GSE22220 clustered as p53-*wt* and p53-*mut* according to p53 status signature expression.

Supplementary Table S1: Common differentially expressed genes among three cell lines upon miR-644a transfection. See Supplementary_Table_S1

Supplementary Table S2: Enrichment of gene sets related to cancer formation and progression of different cancer types in patients with low and high miR-644a signature scores

Name of the gene set	Normalized Enrichment Score (NES)	*p*-value
Gene Sets associated with low miR-644a signature score		
LOPEZ_MESOTELIOMA_SURVIVAL_TIME_UP	−1.499	0.008
WEST_ADRENOCORTICAL_CARCINOMA_VS_ADENOMA_UP	−1.704	0.008
POMEROY_MEDULLOBLASTOMA_PROGNOSIS_DN	−1.714	0.012
MONTERO_THYROID_CANCER_POOR_SURVIVAL_UP	−1.420	0.012
SHEDDEN_LUNG_CANCER_POOR_SURVIVAL_A6	−1.633	0.021
KAUFFMANN_MELANOMA_RELAPSE_UP	−1.543	0.035
MEINHOLD_OVARIAN_CANCER_LOW_GRADE_DN	−1.538	0.037
Gene Sets associated with high miR-644a signature score		
WAMUNYOKOLI_OVARIAN_CANCER_GRADES_1_2_DN	1.683	0.003
WOO_LIVER_CANCER_RECURRENCE_UP	1.480	0.050
LANDIS_BREAST_CANCER_PROGRESSION_DN	1.664	0.013

Supplementary Table S3: Enrichment of gene sets related to metastasis and drug resistance in patients with low and high miR-644a signature scores

Name of the gene set	Normalized Enrichment Score (NES)	*p*-value
**METASTASIS**		
Gene Sets associated with low miR-644a signature score		
WANG_METASTASIS_OF_BREAST_CANCER_ESR1_UP	−1.57	0.032
Gene Sets associated with high miR-644a signature score		
CHANDRAN_METASTASIS_DN	1.494	0.034
**DRUG RESISTANCE**		
Gene Sets associated with low miR-644a signature score		
KANG_DOXORUBICIN_RESISTANCE_UP	−1.557	0.008
KOBAYASHI_EGFR_SIGNALING_24HR_DN	−1.593	0.017
KIM_GASTRIC_CANCER_CHEMOSENSITIVITY	−1.537	0.035
Gene Sets associated with high miR-644a signature score		
RIGGINS_TAMOXIFEN_RESISTANCE_DN	1.452	0.026

Supplementary Table S4: Summary of the GEO datasets used in this study

Dataset	Cancer Type	Study Design	Samples	Array Type	URL
**GSE4922**	Breast	Three breast cancer cohorts (Uppsala (*n* = 249), Stockholm (*n* = 58) and Singapore (*n* = 40)) were reanalyzed for mRNA expression.	289	[HG-U133A] Affymetrix Human Genome U133A Array (*mRNA*)<br>[HG-U133B] Affymetrix Human Genome U133B Array (*mRNA*)	http://www.ncbi.nlm.nih.gov/geo/query/acc.cgi?acc=GSE4922
**GSE2603**	Breast	Subpopulations of MDA-MB-231 that exhibit different metastatic tropisms in immuno-deficient mice together with a cohort of 99 primary breast cancers from different subtypes were profiled.	121	Affymetrix Human Genome U133A Array (*mRNA*)	http://www.ncbi.nlm.nih.gov/geo/query/acc.cgi?acc=gse2603
**GSE16446**	Breast	120 primary ER-negative breast tumors of anthracycline-treated patients were profiled.	120	[HG-U133_Plus_2] Affymetrix Human Genome U133 Plus 2.0 Array (*mRNA*)	http://www.ncbi.nlm.nih.gov/geo/query/acc.cgi?acc=GSE16446
**GSE19536**	Breast	101 breast cancer samples from different subtypes were profiled.	101	Agilent-014850 Whole Human Genome Microarray 4x44K G4112F (Probe Name version) (*mRNA*)<br>Agilent-019118 Human miRNA Microarray 2.0 G4470B (miRNA ID version) (*miRNA*)	http://www.ncbi.nlm.nih.gov/geo/query/acc.cgi?acc=GSE19536
**GSE22220**	Breast	216 and 210 early primary breast tumors from different subtypes were used for mRNA and miRNA profiling respectively. 207 samples from each group were matched.	426	Illumina humanRef-8 v1.0 expression beadchip (*mRNA*)<br>llumina Human v1 MicroRNA expression beadchip (*miRNA*)	http://www.ncbi.nlm.nih.gov/geo/query/acc.cgi?acc=gse22220
**GSE38167**	Breast	31 primary TNBC cases, 13 lymph node metastases and 23 matched normal breast tissues were profiled. 9 cases had matched normal, primary and lymph node metastasis.	67	Agilent-029297 Human miRNA Microarray (Feature Number version) (*miRNA*)	http://www.ncbi.nlm.nih.gov/geo/query/acc.cgi?acc=gse38167
**GSE40059**	Breast	10 breast cancer cell lines (4 agressive and 6 less agressive) and 2 immortalized breast epithelium cell lines were profiled.	12	[HG-U133_Plus_2] Affymetrix Human Genome U133 Plus 2.0 Array (*mRNA*)<br>[miRNA-1_0] Affymetrix miRNA Array (miRNA)	http://www.ncbi.nlm.nih.gov/geo/query/acc.cgi?acc=GSE40059
**GSE45666**	Breast	101 breast tumors from diffferent subtypes and 15 matched adjacent breast normal tissue samples were profiled.	116	Agilent-021827 Human miRNA Microarray G4470C (Feature Number version) (*miRNA*)	http://www.ncbi.nlm.nih.gov/geo/query/acc.cgi?acc=gse45666
**GSE58606**	Breast	11 normal breast and 122 breast cancer samples from different subtypes were profiled.	133	miRCURY LNA microRNA Array 7th generation - human, mouse & rat [product# 208502] (miRBase 18.0) (*miRNA*)	http://www.ncbi.nlm.nih.gov/geo/query/acc.cgi?acc=GSE58606
**GSE58644**	Breast	321 samples from breast cancer patients from different subtypes were profiled.	321	[HuGene-1_0-st] Affymetrix Human Gene 1.0 ST Array [transcript (gene) version] (*mRNA*)	http://www.ncbi.nlm.nih.gov/geo/query/acc.cgi?acc=GSE58644
**METABRIC project**	Breast	1,302 breast tumors with detailed clinical annotation and long-term follow-up were profiled for mRNA and miRNA expression.	1302	Illumina HT 12<br>(*mRNA*) Agilent ncRNA 60k (*miRNA*)	https://www.ebi.ac.uk/ega/studies/EGAS00000000122
**KM Plotter**	Breast Ovarian	This online database can access patient data from several public datasets of breast, ovarian, lung and gastric cancer	558 Breast and 528 Ovarian (with known p53 status)	Affymetrix HG-U133A, HG-U133 Plus 2.0 and HG-U133A 2.0 (*mRNA*)	http://kmplot.com/analysis/index.php?p=background

Supplementary Table S5: Sequences of forward and reverse primers used in qRT-PCR analysis

Gene Symbol	Gene ID	Forward Primer	Reverse Primer
CTBP1	1487	5′-acccttacttgtcggatggc-3′	5′-atgaggtggtggttgtgct-3′
CDH1	999	5′-cccgggacaacgtttattac-3′	5′-gctggctcaagtcaaagtcc-3′
KRT18	3875	5′-tgatgacaccaatatcacacga-3′	5′-ggcttgtaggccttttacttcc-3′
ZO1	7082	5′-cagagccttctgatcattcca-3′	5′-catctctactccggagactgc-3′
ZEB1	6935	5′-gggaggagcagtgaaagaga-3′	5′-tttcttgcccttcctttctg-3′
ZEB2	9839	5′-aagccagggacagatcagc-3′	5′-ccacactctgtgcatttgaact-3′
FN	2335	5′-ctggccgaaaatacattgtaaa-3′	5′-ccacagtcgggtcaggag-3′
SNAI2	6591	5′-tggttgcttcaaggacacat-3′	5′-gttgcagtgagggcaagaa-3′
MMP9	4318	5′-gaaccaatctcaccgacagg-3′	5′-gccacccgagtgtaaccata-3′
NOXA	5366	5′-atgaatgcaccttcacattcctct-3′	5′-tccagcagagctggaagtcgagtgt-3′
p21	1026	5′-tgagccgcgactgtgatg-3′	5′-gtctcggtgacaaagtcgaagtt-3′
p53	7157	5′-cccaagcaatggatgatttga-3′	5′-ggcattctgggagcttcatct-3′
BAX	581	5′-gggtggttgggtgagactc-3′	5′-agacacgtaaggaaaacgcatta-3′
HPRT	3251	5′-tgaccttgatttattttgcatacc-3′	5′-cgagcaagacgttcagtcct-3′
ACTB	60	5′-ccaaccgcgagaagatga-3′	5′-ccagaggcgtacagggatag-3′
GAPDH	2597	5′-gcccaatacgaccaaatcc-3′	5′-agccacatcgctcagacac-3′
MYC	4609	5′-cagctgcttagacgctggatttt-3′	5′-accgagtcgtagtcgaggtcat-3′
PUMA	27113	5′-ccagggctgcttccacgacg-3′	5′-acactgccgagggcaccagg-3′

Supplementary Table S6: List of antibodies used in Western blot analysis

Antibody	Firm	Catalog number
CTBP1	BD	612042
Beta-actin	MP Biomedicals	691001
Cleaved Caspase 3	Cell Signaling Technology	9661
CDK2	Sigma	C5223
CDK4	Epitomics	2341-1
Cyclin D1	Cell Signaling Technology	2922
Phospho-Rb (S807/811)	Cell Signaling Technology	8516
p53	Cell Signaling Technology	2527
p21	BD Biosciences	554228
Noxa	Santa Cruz Biotechnology	SC-30209
p-Cdc2	Cell signaling Technology	2543S
p-Cdc25C	Cell signaling Technology	4901S
ZO1	Cell Signaling Technology	13663
Fibronectin	Santa Cruz Biotechnology	SC-81767
ZEB-1	Cell Signaling Technology	3396
Vimentin	Cell Signaling Technology	5741
