# Supplementary material for: The miR-644a/CTBP1/p53 axis suppresses drug resistance by simultaneous inhibition of cell survival and epithelial-mesenchymal transition in breast cancer
Source: Oncotarget. 2016 Jul 8;7(31):49859–77. doi: 10.18632/oncotarget.10489 (PMC5226553; doi:10.18632/oncotarget.10489)
Supplement: Supplementary file 2 [file oncotarget-07-49859-s002.docx]

**Supplementary Table 1. Common differentially expressed genes among three cell lines upon miR-644a transfection.**

| Genes downregulated by miR-644a | | | | | | | | |
| --- | --- | --- | --- | --- | --- | --- | --- | --- |
| ABCD1 | CNOT6L | GC | | | MED16 | PSORS1C2 | SMAD5 | VPS28 |
| ACLY | COQ9 | GDE1 | | | MED20 | PTP4A1 | SMAP2 | VTI1B |
| ACOT7 | CORO1C | GGA2 | | | MED28 | PXDN | SNX3 | XRN1 |
| ACTG1 | COX10 | GLB1 | | | MEF2D | PYCR1 | SNX5 | ZADH2 |
| ADCY9 | CPNE1 | GLT25D1 | | | MEST | RAB11FIP5 | SPDEF | ZDHHC18 |
| ADIPOR2 | CREB3L2 | GLTP | | | MGLL | RAB15 | SPRED2 | ZMAT5 |
| AHCYL1 | CRTAP | GMPPA | | | MIF4GD | RAB22A | SPRY4 | ZMIZ1 |
| ALDH4A1 | CSF1R | GNG7 | | | MKI67 | RAB28 | SPTLC1 | ZNF323 |
| ANKRD17 | CTBP1 | GXYLT1 | | | MPV17 | RAC1 | SPTLC2 | ZNF35 |
| AP3M1 | CTDSPL | H2AFX | | | MTX1 | RALBP1 | SRA1 | ZSCAN5A |
| APEH | CYBASC3 | H2AFY | | | MXD1 | RARA | SSH3 |  |
| APP | DALRD3 | HDHD1 | | | MYB | RBM15B | SSR1 |  |
| ARHGAP1 | DARS | HM13 | | | NCOR2 | RCE1 | SSR2 |  |
| ARPC4 | DBT | HMG20B | | | NDST1 | RHOBTB2 | ST3GAL1 |  |
| ARPC5 | DCI | HNRPDL | | | NEK6 | RIC8A | STRADB |  |
| ARPP19 | DDHD2 | HPS3 | | | NFYC | RNF130 | STX6 |  |
| ASAP1 | DDIT3 | HSD3B7 | | | NPLOC4 | RNF4 | SUOX |  |
| ASCC2 | DDR1 | HSF1 | | | NPM3 | RPL23A | SYNCRIP |  |
| ATF4 | DENND1A | HSPBP1 | | | NR1H3 | RPL32 | TCEB2 |  |
| ATOX1 | DHCR24 | ICK | | | NRBF2 | RPS28 | TDP1 |  |
| ATP1A1 | DIDO1 | ICMT | | | NSA2 | RPS6KA3 | TFAP2A |  |
| ATP6V1C1 | DNASE1L1 | IFI30 | | | OSBPL5 | RRP36 | TIMM22 |  |
| ATP8B2 | DPM2 | IMPAD1 | | | PAFAH1B1 | RTN3 | TMEM117 |  |
| ATPIF1 | DSCR3 | INPP5A | | | PAICS | RTN4 | TNPO1 |  |
| ATXN1 | ECE1 | INSIG2 | | | PDIA4 | RUFY1 | TOLLIP |  |
| BCKDK | EDDM3B | IPO11 | | | PDXK | S100A16 | TOR1A |  |
| BCL7B | EFHD1 | IQGAP1 | | | PEF1 | SAMM50 | TRIB3 |  |
| BCS1L | EIF2B1 | IRX5 | | | PERP | SCAMP3 | TRIOBP |  |
| BIRC5 | EIF2S3 | ITGB1BP1 | | | PFKFB3 | SEC16A | TSHZ1 |  |
| BMPR2 | EIF3F | ITGB3 | | | PHF19 | SEC24D | TTLL5 |  |
| BNIP1 | EIF4B | IYD | | | PHLDA2 | SEL1L | TUBA3D |  |
| C11orf75 | ELAC2 | KCTD1 | | | PHPT1 | SEPN1 | TWSG1 |  |
| C17orf70 | ELAVL1 | KCTD20 | | | PIN1 | 2-Sep | TXNDC5 |  |
| C19orf62 | ENAH | KDELR3 | | | PIR | SFT2D3 | UBE2D4 |  |
| CALU | ERGIC1 | KIAA1191 | | | PKP4 | SFXN2 | UBE2L3 |  |
| CAMK2D | ERGIC2 | KIF20A | | | PLAGL2 | SH3BP4 | UBE2N |  |
| CAPZB | ESPN | KLK3 | | | PLOD1 | SH3GL1 | UBE4B |  |
| CC2D1A | EVL | KPNA6 | | | PODXL | SIRT5 | UGDH |  |
| CD164 | EXOC4 | LARS2 | | | POMT2 | SLC12A8 | UGGT1 |  |
| CDC42EP1 | FAF1 | LEPREL4 | | | PON2 | SLC25A22 | UGT3A1 |  |
| CDC42SE1 | FANCG | LIMK1 | | | PPFIBP1 | SLC25A23 | UQCR10 |  |
| CDK6 | FARP1 | LIN54 | | | PPIL1 | SLC25A44 | URM1 |  |
| CEP55 | FGFRL1 | LMNB2 | | | PPP1R7 | SLC2A1 | UROS |  |
| CEP57 | FOXK1 | LRTOMT | | | PPP2R4 | SLC30A5 | UXT |  |
| CHM | FOXM1 | LTBR | | | PRKCA | SLC31A1 | VIPAR |  |
| CHMP7 | GABBR1 | MAP1S | | | PRUNE2 | SLC40A1 | VKORC1 |  |
| CHP | GALNT1 | MAPK9 | | | PSMA4 | SLC44A1 | VMA21 |  |
| CNOT1 | GAPDH | MARVELD2 | | | PSMD7 | SLC9A1 | VPS18 |  |
| Genes upregulated by miR-644a | | | |  |  |  |  |  |
| AAK1 | FYN | | UHMK1 |  |  |  |  |  |
| ABL1 | GABRG2 | | USP16 |  |  |  |  |  |
| ABTB1 | GADD45B | | VHL |  |  |  |  |  |
| ACSM3 | GATAD2B | | WDR12 |  |  |  |  |  |
| ALDH1A3 | GCA | | YOD1 |  |  |  |  |  |
| AMFR | GDF15 | | YTHDF2 |  |  |  |  |  |
| ANKRA2 | GLRX | | YWHAB |  |  |  |  |  |
| ANXA1 | GRPR | | ZCCHC11 |  |  |  |  |  |
| ANXA3 | HABP4 | | ZMAT3 |  |  |  |  |  |
| AP4B1 | HBP1 | | ZNF224 |  |  |  |  |  |
| ARFGEF2 | HSPB8 | | ZNF326 |  |  |  |  |  |
| ARHGEF19 | IGF1R | | ZSCAN2 |  |  |  |  |  |
| ARL5B | IMPA1 | |  |  |  |  |  |  |
| ATP5S | INPP4B | |  |  |  |  |  |  |
| ATP6V0A1 | IPPK | |  |  |  |  |  |  |
| ATXN3 | IRS2 | |  |  |  |  |  |  |
| BEX2 | ITPR1 | |  |  |  |  |  |  |
| BMF | KIFAP3 | |  |  |  |  |  |  |
| BNIP3L | LAMB2 | |  |  |  |  |  |  |
| BRE | LOC399491 | |  |  |  |  |  |  |
| C10orf46 | LTB4R2 | |  |  |  |  |  |  |
| C5orf41 | MAFB | |  |  |  |  |  |  |
| CABC1 | MAGI3 | |  |  |  |  |  |  |
| CAPRIN2 | MCL1 | |  |  |  |  |  |  |
| CELF1 | MCTP1 | |  |  |  |  |  |  |
| CFH | MDN1 | |  |  |  |  |  |  |
| CGN | MICALCL | |  |  |  |  |  |  |
| CHST14 | MNAT1 | |  |  |  |  |  |  |
| CITED4 | MT1F | |  |  |  |  |  |  |
| COL4A3BP | MT1H | |  |  |  |  |  |  |
| COPG2 | MYH14 | |  |  |  |  |  |  |
| CREB5 | NAA35 | |  |  |  |  |  |  |
| CYP4B1 | NACC2 | |  |  |  |  |  |  |
| CYR61 | NARS2 | |  |  |  |  |  |  |
| DCAF4 | NDUFB4 | |  |  |  |  |  |  |
| DCAF7 | NFYA | |  |  |  |  |  |  |
| DCLK2 | NHP2L1 | |  |  |  |  |  |  |
| DCUN1D3 | NKRF | |  |  |  |  |  |  |
| DGUOK | NME7 | |  |  |  |  |  |  |
| DHRS3 | NPAT | |  |  |  |  |  |  |
| DUT | NPR3 | |  |  |  |  |  |  |
| ELF4 | NR2C1 | |  |  |  |  |  |  |
| ENTPD4 | NTN4 | |  |  |  |  |  |  |
| ETF1 | OFD1 | |  |  |  |  |  |  |
| EWSR1 | OTUD4 | |  |  |  |  |  |  |
| EXOSC5 | PALLD | |  |  |  |  |  |  |
| FAM13A | PANK3 | |  |  |  |  |  |  |
| FAM3C | PARD3 | |  |  |  |  |  |  |
| FBXO10 | UBE2H | |  |  |  |  |  |  |
| FHOD3 | UGCG | |  |  |  |  |  |  |
